# Tracking the immune response by MRI using biodegradable and ultrasensitive microprobes

**DOI:** 10.1126/sciadv.abm3596

**Published:** 2022-07-13

**Authors:** Sara Martinez de Lizarrondo, Charlene Jacqmarcq, Mikael Naveau, Manuel Navarro-Oviedo, Swannie Pedron, Alexandre Adam, Barbara Freis, Stephane Allouche, Didier Goux, Sarah Razafindrakoto, Florence Gazeau, Damien Mertz, Denis Vivien, Thomas Bonnard, Maxime Gauberti

**Affiliations:** ^1^ Normandie Université, UNICAEN, INSERM, PhIND (Physiopathology and Imaging of Neurological Disorders), Institut Blood and Brain @ Caen-Normandie, Cyceron, 14000 Caen, France.; ^2^ Normandie Université, UMS 3408 Cyceron, CNRS, University of Caen Normandy, GIP CYCERON, Caen, France.; ^3^ Institut de Physique et de Chimie des Matériaux de Strasbourg (IPCMS), UMR-7504 CNRS—Université de Strasbourg, 23 rue du Lœss, 67034 Strasbourg, France.; ^4^ CHU Caen, Department of Biochemistry, CHU de Caen Côte de Nacre, Caen, France.; ^5^ Centre de Microscopie Appliquée à la Biologie (CMAbio), UniCaen, Normandie University, SF4206 Icore, 14000 Caen, France.; ^6^ MSC, Université de Paris CNRS, UMR7057, 45 rue des Saints Pères 75006, Paris, France.; ^7^ CHU Caen, Clinical Research Department, CHU de Caen Côte de Nacre, Caen, France.; ^8^ CHU Caen, Department of Diagnostic Imaging and Interventional Radiology, CHU de Caen Côte de Nacre, Caen, France.

## Abstract

Molecular magnetic resonance imaging (MRI) holds great promise for diagnosis and therapeutic monitoring in a wide range of diseases. However, the low intrinsic sensitivity of MRI to detect exogenous contrast agents and the lack of biodegradable microprobes have prevented its clinical development. Here, we synthetized a contrast agent for molecular MRI based on a previously unknown mechanism of self-assembly of catechol-coated magnetite nanocrystals into microsized matrix-based particles. The resulting biodegradable microprobes (M3P for microsized matrix-based magnetic particles) carry up to 40,000 times higher amounts of superparamagnetic material than classically used nanoparticles while preserving favorable biocompatibility and excellent water dispersibility. After conjugation to monoclonal antibodies, targeted M3P display high sensitivity and specificity to detect inflammation in vivo in the brain, kidneys, and intestinal mucosa. The high payload of superparamagnetic material, excellent toxicity profile, short circulation half-life, and widespread reactivity of the M3P particles provides a promising platform for clinical translation of immuno-MRI.

## Teaser

Targeted microparticles of polydopamine and iron oxide reveal site of leukocyte trafficking on magnetic resonance images.

## INTRODUCTION

Magnetic resonance imaging (MRI) is a promising modality for molecular imaging but remains limited by low sensitivity (*1*). The usual concentration of relevant targets for molecular imaging is around 10^−9^ to 10^−12^ M in human tissues, whereas MRI detects clinically approved gadolinium chelate at concentrations over 10^−6^ M (*2*, *3*). Magnetic resonance spectroscopy and deuterium metabolic imaging allow quantitative mapping of the in vivo dynamics of cellular metabolism but are not available for all molecular targets (*4*). Amplification strategies aiming at binding a large amount of contrast material to the molecular target are thus necessary. To date, nanosized contrast agents such as ultrasmall particles of iron oxide (USPIO) with a diameter ranging from 10 to 50 nm have been the primary focus of molecular MRI studies (*5*). USPIO can be conjugated to targeting moieties such as peptides or antibodies and have a favorable safety profile in humans. However, the low sensitivity (due to the small amount of iron payload per particle), poor specificity (due to passive extravasation through permeated endothelial barriers), and long delay between administration and imaging (up to 24 hours after intravenous injection) have precluded the use of USPIO as targeted molecular imaging agents (*6*).

More recently, microparticles of iron oxide (MPIO) with diameters close to 1 μm have been used as a new family of contrast agent for molecular MRI (*7*). MPIO display a higher sensitivity than USPIO thanks to higher iron content (*8*). We and others demonstrated the applicability of targeted MPIO for molecular MRI in several experimental models, including cardiovascular (*9*, *10*) and neurovascular disorders (*11*), autoimmune diseases (*12*, *13*), and cancer (*14*). Notably, MPIO are rapidly eliminated from the circulation by the reticuloendothelial system, thereby limiting their ability to reach their target (*15*). Therefore, there are strict constrains on the inner structure and coating of MPIO to allow their accumulation at concentrations high enough to be detected by MRI. Unfortunately, the MPIO used in preclinical studies are made of a polystyrene matrix and are not clinically compatible (*6*). Covalent assembly of USPIO with peptidase-degradable bonds has been described as an alternative to MPIO, but the resulting product has a low sensitivity (*16*). Thus, there is still no contrast agent combining the high sensitivity of currently available MPIO with the biocompatibility and biodegradability of USPIO. This limitation prevents the clinical translation of molecular MRI.

Here, we describe the production and characterization of a new class of contrast agent based on a previously unknown mechanism of self-assembly of dopamine-coated magnetite nanocrystals (MNcs) into microsized matrix-based magnetic particles (M3P). Using only three common reagents (iron chloride, dopamine, and ammonia), we produced M3P with mean diameters tunable from 300 to 700 nm and polydispersity index <0.2. Thanks to a biocompatible, hydrophilic, and reactive polydopamine (PDA) matrix (*17*, *18*), M3P can be efficiently functionalized with targeting moieties such as monoclonal antibodies (immuno-MRI). By targeting vascular cell adhesion molecule–1 (VCAM-1) (*19*) and mucosal addressin cell adhesion molecule–1 (MAdCAM-1) (*20*), two proteins expressed by activated endothelial cells and involved in leucocyte trafficking, M3P allows tracking the immune response in a noninvasive manner. We demonstrate the applicability of this new platform for ultrasensitive molecular imaging of inflammation in the brain, kidneys, and intestinal mucosa.

## RESULTS

### Synthesis of M3P

**Fig. 1.**
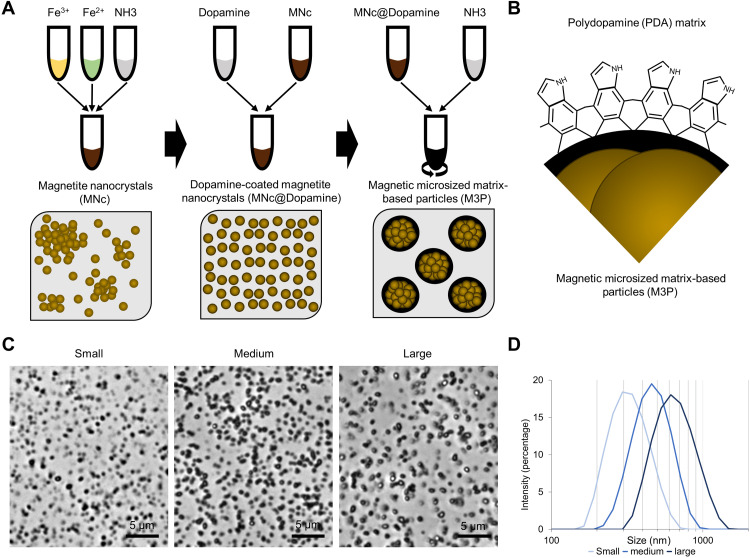
Synthesis of M3P. (**A**) Schematic representation of the main step of M3P synthesis. First, MNc were produced by coprecipitation of ferric (Fe^3+^) and ferrous chloride (Fe^2+^) in the presence of ammonia (NH_3_). Second, MNc were stabilized by a coating with dopamine (MNc@Dopamine). Third, PDA matrix was initiated by the addition of different doses of ammonia under mechanical stirring, leading to the formation of M3P. (**B**) Schematic representation of the surface and inner structure of M3P. (**C**) Representative phase microscopy images of production of M3P of different sizes obtained by using different concentrations of ammonia during synthesis. (**D**) Size distribution of M3P as assessed by dynamic light scattering (representative of three independent experiments in three independent batches).

Our first objective was to produce a microsized magnetic particle that is fully biodegradable and can be functionalized for molecular MRI. Previous studies demonstrated that in alkaline buffers, dopamine oxidation induces the formation of submicrometric particles of PDA (*21*). We hypothesized that nanocrystals coated with dopamine would be incorporated as building blocks during the formation of PDA particles. Thus, we synthetized MNc by a classical coprecipitation method using a 2:1 FeCl_3_:FeCl_2_ ratio (Fig. 1A and fig. S1). After several washing steps, purified nanocrystals (MNc) were incubated with dopamine under continuous sonication to obtain dopamine-coated MNc (MNc@Dopamine). Dynamic light scattering data on MNc@Dopamine are presented in fig. S2. The resulting suspension was stirred using a mechanical disperser at room temperature, and ammonia was added to start dopamine polymerization. This led to the self-assembly of catechol-coated MNc into large multimeric particles (M3P; Fig. 1B). The mean hydrodynamic diameters of the M3P can be tuned from 300 to 700 nm (as measured by dynamic light scattering) by adjusting the concentration of ammonia during particle formation. Polydispersity indexes were all inferior to 0.2 (Fig. 1, C and D), and zeta potentials at pH 7.4 ranged from −30 to −42 mV. Dynamic light scattering data on large 700-nm M3P are presented in fig. S3. M3P were highly dispersible in water, without noticeable aggregate, as assessed by optical phase microscopy at different scales (movies S1 and S2). The particles produced were large enough to be rapidly separated from an aqueous solution using a bench magnet (fig. S4).

### Physical characterization of M3P

**Fig. 2.**
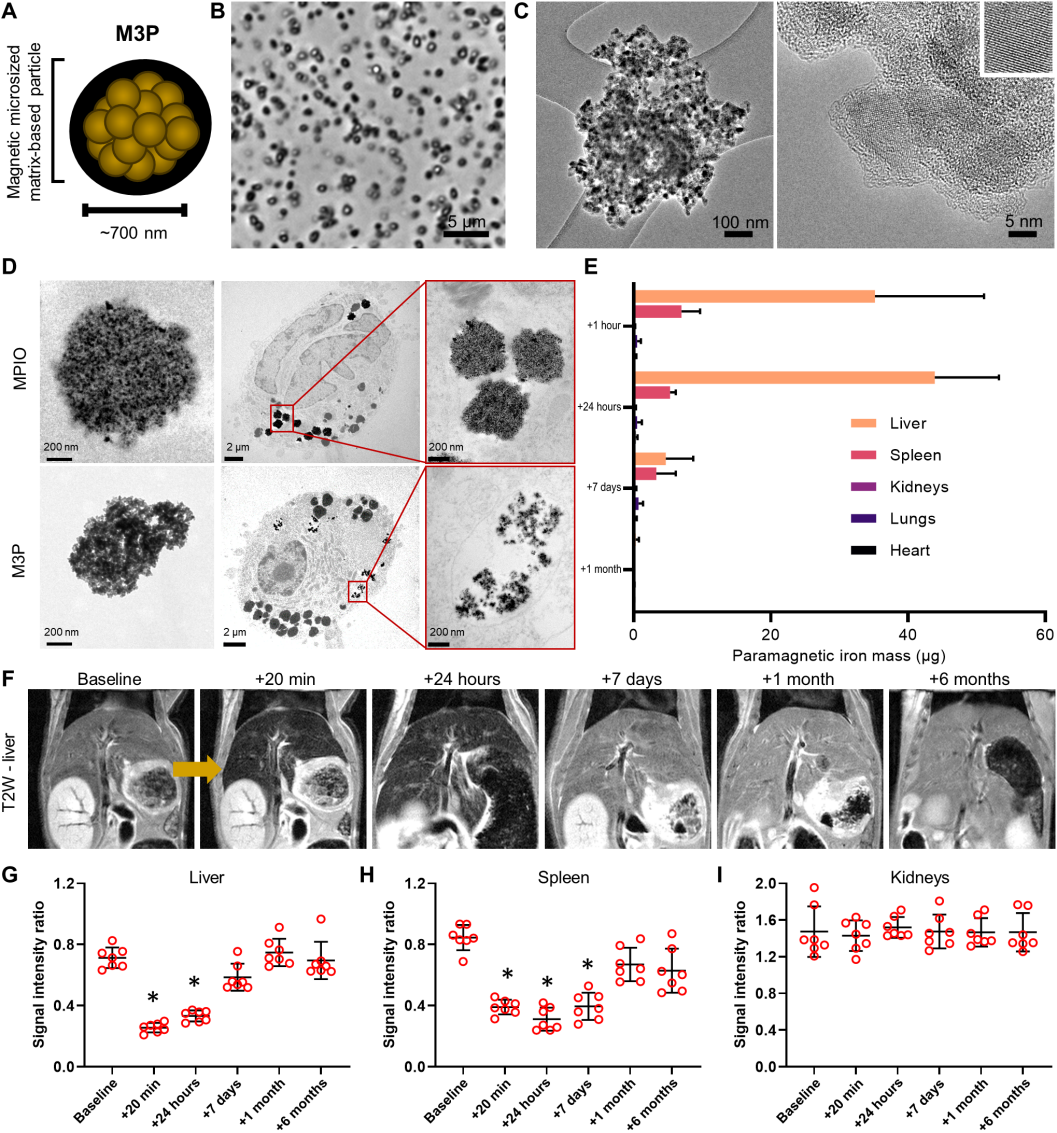
Large M3P are degraded in vitro and in vivo. (**A**) Schematic representation of large M3P. (**B**) Representative phase microscopy image of one batch of large M3P. (**C**) Representative high-resolution transmission electron microscopy (TEM) of a large M3P. The small insert on the upper right depicts the crystalline structure of one clustered MNc. (**D**) Representative TEM images of commercial MPIO (top) and M3P (down) before (left) and 96 hours (middle and right) after incubation with macrophages. (**E**) Mass of paramagnetic iron in the liver, spleen, kidneys, lungs, and heart at different time points after intravenous injection of large M3P (4 mg/kg), as assessed by electronic paramagnetic resonance. (**F**) Representative longitudinal T2-weighted images of the liver at baseline and at different time points after intravenous injection of large M3P (4 mg/kg). (**G**) Evolution of the signal intensity on T2-weighted imaging in the liver (*n* = 7 per group). Ratio was calculated by taking the signal of the paravertebral muscles as reference. (**H**) Evolution of the signal intensity on T2-weighted imaging in the spleen (*n* = 7 per group). (**I**) Evolution of the signal intensity on T2-weighted imaging in the right kidney (*n* = 7 per group). *P < 0.05 versus baseline.

The large 700-nm M3P, carrying the largest amount of contrast material, were further characterized by optical and electronic microscopy (Fig. 2, A to C). These particles have pseudo-spherical shapes and are made of numerous MNcs embedded in a matrix with comparatively low electronic density, consistent with a PDA matrix. The respective amounts of the organic fraction (PDA) and the inorganic fraction (magnetite) composing the M3P were quantified by thermogravimetric analysis (TGA), revealing that 67.3% of the particle mass is inorganic (fig. S5). X-ray powder diffraction patterns of the particles exhibited the characteristic peaks of a spinel crystalline structure with a mean crystallite size of 11.0 nm, with lattice parameters between magnetite and maghemite phases (fig. S6). Magnetization curves at 5 and 300 K are presented in fig. S7. At 5 K, a hysteresis cycle can be observed when, at 300 K, the particles present a superparamagnetic behavior. Saturation magnetization was 65.3 electromagnetic unit/g as assessed by superconducting quantum interference device magnetometry. Smaller M3P presented similar characteristics (figs. S5 and S6). At 37°C and 1.41 T (60 MHz), the relaxivity r1 value of 700-nm M3P was low (2.1 mM^−1^ s^−1^), whereas the r2 value was high (188.2 mM^−1^ s^−1^), as assessed by a magnetic resonance spectrometer. Using a preclinical MRI, at room temperature and 7 T (300 MHz), the relaxivity values for r1, r2, and r2* were 0.35, 139.9, and 301.7 mM^−1^ s^−1^, respectively (fig. S8). All these parameters are within the range of previously reported values in the literature for superparamagnetic particles with similar crystallite sizes, supporting that clustering MNc using PDA preserves their favorable superparamagnetic properties.

### Biodegradability of M3P

Biodegradability of 700-nm M3P was investigated both in vitro and in vivo. First, the particles were incubated in phosphate-buffered saline (PBS), artificial lysosomal fluid (ALF), citrate buffer, or citrate buffer with hydrogen peroxide. These buffers mimic the lysosomal environment where large particles accumulate after intravenous injection. Degradation was monitored by direct visual inspection and ultraviolet-visible (UV-vis) spectroscopy for 1 week at 37°C under mild agitation (fig. S9). We studied the degradation of M3P of different sizes and MNc@Dopamine (i.e., without PDA). Visual aspect and UV-vis spectroscopy of MNc@Dopamine and M3P in PBS did not change substantially during the monitoring. In ALF and citrate buffers, the brown color of the MNc@Dopamine solution turned with time into the expected yellow color of free Fe(III) ions. The same phenomenon occurred for M3P solutions, but an additional dark precipitate remained present at every time point. This precipitate appeared dark brown on bright-field microscopy and was not attracted by a magnet (fig. S10). This appearance was compatible with free PDA remaining after MNc degradation. In line with this hypothesis, this precipitate progressively solubilized in samples containing hydrogen peroxide, which generates hydroxyl radicals that are able to degrade PDA. UV-vis spectroscopy further supported these findings by showing progressive degradation of M3P in ALF and citrate, with persistence of a chemical species absorbing in the near-infrared region (650 nm), compatible with PDA (fig. S8, B to D). In citrate with hydrogen peroxide buffer, the absorbance of the solution initially containing M3P progressively diminished in the near-infrared region until reaching zero after 7 days of incubation. At day 7, the UV-vis curve was almost undistinguishable between solutions initially containing dopamine-coated iron oxide nanocrystals or M3P, supporting complete degradation of PDA in this buffer mimicking conditions of oxidative stress by endogenous reactive oxygen species.

Second, the degradation of 700-nm M3P was investigated in cell culture of macrophages, the cell type in which large particles accumulate after intravenous injection in vivo. Macrophages were obtained by activation of a human monocytic cell line (THP-1) and were incubated with either M3P or nonbiodegradable commercial MPIO (Dynabeads MyOne) made of iron oxide nanocrystals embedded in a polystyrene matrix. After 96 hours, the cells and particles were observed by transmission electronic microscopy (Fig. 2D). Both types of particles were internalized at this time point. Whereas commercial MPIO remained morphologically intact, M3P fragmented into smaller particles, demonstrating that the PDA matrix is rapidly degraded and releases iron oxide nanocrystals once internalized in macrophages.

In vivo, the biodistribution of M3P was investigated in mice at different time points after intravenous injection (from 1 hour to 6 months) by electron paramagnetic resonance (EPR) that quantifies superparamagnetic components in ex vivo tissue (Fig. 2E and fig. S11), in vivo MRI (Fig. 2, F to I, and fig. S12), and histochemistry with Prussian blue staining (fig. S13). All the methods concur in showing that M3P first accumulate in the liver and spleen (reticuloendothelial system) and that their superparamagnetic components are subsequently degraded or excreted. No residual particles were detected more than 7 days after intravenous injection. There was no significant accumulation of M3P in the kidneys, lungs, or heart at any time points (fig. S13). Neither microvascular plugging nor pathological change in liver volume was observed (fig. S14).

### Biocompatibility of M3P

The biocompatibility of 700-nm M3P was investigated both in vitro and in vivo. No significant cytotoxicity on endothelial cells [human umbilical cord endothelial cells (HUVECs)] was detected at doses up to 320 μg/ml for 3 hours (figs. S15 and S16). After 24 hours of incubation, the viability of endothelial cells was slightly reduced in the high-dose groups. Hemolysis (fig. S17), coagulation (fig. S18), and fibrinolysis (figs. S19 and S20) were all unaffected by 700-nm M3P using either human or murine blood. After intravenous injection of 700-nm M3P (4 mg/kg; equivalent iron), body weight remained within normal ranges (fig. S21) and neither liver enzyme (fig. S22), inflammatory cytokines (fig. S23), nor histological findings (fig. S24) were of toxicological significance.

### Conjugation of M3P to monoclonal antibodies for targeted imaging

**Fig. 3.**
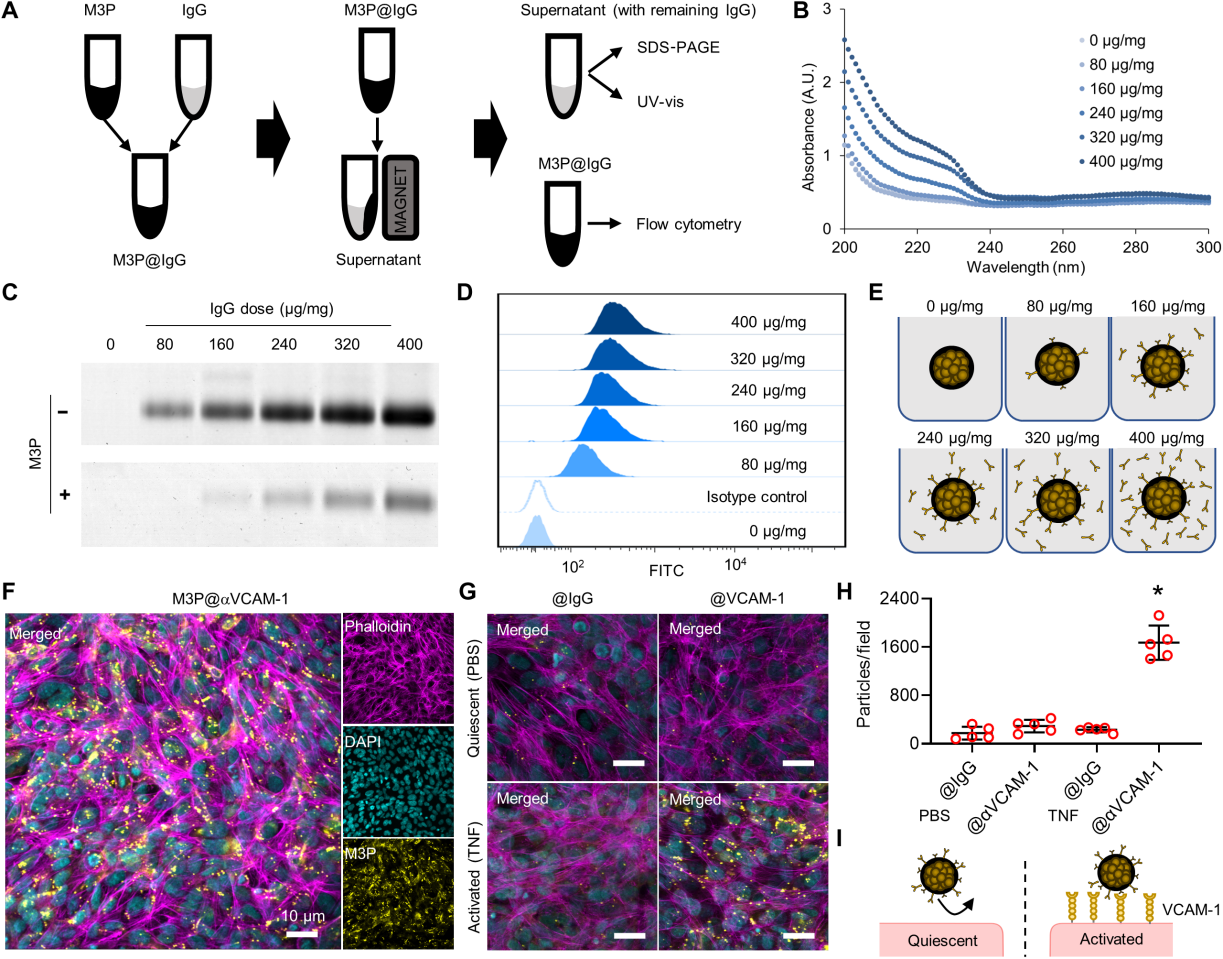
Complexation of immunoglobulins to M3P. (**A**) Schematic representation of the methods for complexation and characterization of M3P. (**B**) UV-vis spectroscopy of the supernatant of M3P@IgG after complexation to different doses of IgG (from 80 to 400 μg/mg). A.U., arbitrary units. (**C**) SDS-PAGE of the supernatant of M3P@IgG after complexation to different doses of IgG. (**D**) Flow cytometry of M3P@IgG after complexation to different doses of IgG (rat) using anti-rat or control (anti-goat) secondary antibodies. (**E**) Schematic representation of M3P@IgG and its supernatant according to the dose of IgG used during complexation. (**F**) Representative immunofluorescence images of anti-human M3P@αVCAM-1 after incubation with activated human cerebral endothelial cells (hCMECD/3). (**G**) Representative immunofluorescence images of anti-human M3P@αVCAM-1 or control M3P@IgG after incubation with quiescent (treated with PBS) or activated (treated with TNF) hCMECD/3. (**H**) Corresponding quantification of the number of particles per field (*n* = 5 per group). *P < 0.05 versus all other groups. (**I**) Schematic representation of the main findings revealing selective binding of M3P@αVCAM-1 on activated human endothelial cells. DAPI, 4′,6-diamidino-2-phenylindole.

Having demonstrated favorable biocompatibility and biodegradability profiles, we investigated the feasibility of using 700-nm M3P as a platform for immuno-MRI. To this aim, we coated M3P with antibodies in phosphate buffer at pH 8.5 since alkaline buffer favors reactive quinone over catechol groups on the PDA coating. We performed a dose-response experiment by varying the concentration of antibodies [immunoglobulin G (IgG)] in the solution during coupling to obtain antibody/particle ratio between 0 and 400 μg/mg (Fig. 3A). Then, we measured the concentration of bound antibodies on M3P by flow cytometry and the concentration of unbound antibodies in the solution by SDS–polyacrylamide gel electrophoresis (SDS-PAGE) and UV-vis (Fig. 3, B to D). We selected the dose of 160 μg of antibodies for 1 mg of M3P for further experiments since the yield of coupling decreased at higher doses (Fig. 3, D and E). Using this ratio, we coated M3P with monoclonal antibodies targeting mouse VCAM-1, MAdCAM-1, or control IgG (M3P@αVCAM-1, M3P@αMAdCAM-1, and M3P@IgG, respectively). Using fluorescent secondary antibodies, the conjugated M3P can be detected by immunofluorescence by revealing the primary antibodies on their surface (fig. S25), confirming the success of the coating step.

Then, we investigated the binding of targeted M3P in vitro. To this aim, anti-human M3P@αVCAM-1 or control M3P@IgG was incubated with either quiescent or activated human cerebral endothelial cells (hCMECD/3). The number of bound particles was evaluated by immunofluorescence microscopy. M3P@αVCAM-1 bound significantly more to activated endothelial cells than control M3P@IgG (Fig. 3, F to H). Moreover, M3P@αVCAM-1 bound significantly more to activated than quiescent endothelial cells, in line with a higher expression of VCAM-1 after tumor necrosis factor (TNF) stimulation by the formers as assessed by flow cytometry (fig. S26). These results demonstrate that M3P coated with anti–VCAM-1 monoclonal antibodies are able to bind selectively activated human endothelial cells (Fig. 3I).

### M3P@αVCAM-1 reveals neuroinflammation at high sensitivity

**Fig. 4.**
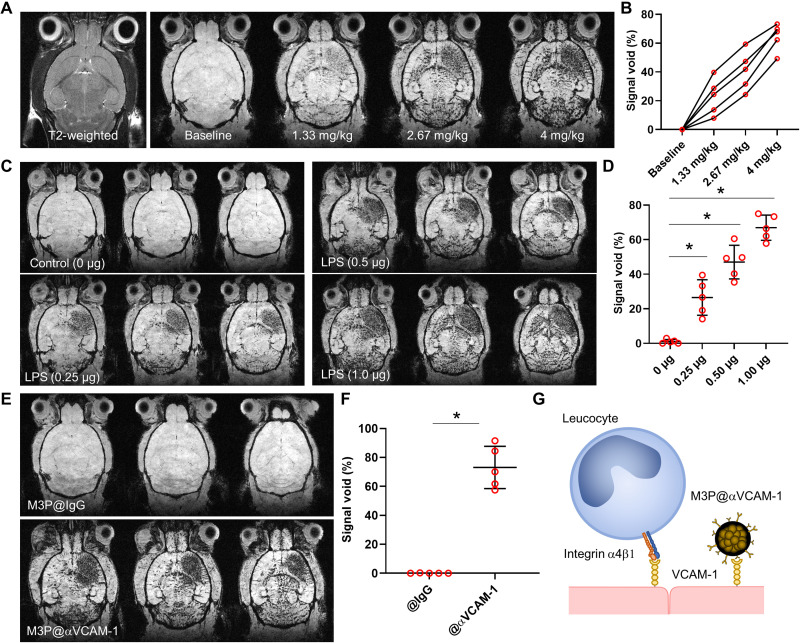
Ultrasensitive molecular imaging of VCAM-1 in the inflamed brain. (**A**) Representative T2-weighted image and T2*-weighted images at baseline and 10 min after successive intravenous injection of M3P targeted against VCAM-1 using monoclonal antibodies (M3P@αVCAM-1) up to 4 mg/kg (equivalent iron) 24 hours after injection of 1 μg of LPS in the right striatum. (**B**) Corresponding quantification of M3P@αVCAM-1 induced signal void in the right striatum (*n* = 5). (**C**) Representative T2*-weighted images (three slices per animal) after intravenous injection of M3P@αVCAM-1 (4 mg/kg) 24 hours after injection of different doses of LPS in the right striatum (from 0 to 1.0 μg). (**D**) Corresponding quantification of M3P@αVCAM-1 induced signal void in the right striatum (*n* = 5 per group). (**E**) Representative T2*-weighted images (three slices per animal) after intravenous injection of M3P@IgG or M3P@αVCAM-1 (4 mg/kg) 24 hours after injection of 1 μg of LPS in the right striatum. (**F**) Corresponding quantification of signal void in the right striatum (*n* = 5 per group). (**G**) Schematic representation of the findings that M3P@αVCAM-1 binds VCAM-1 on the surface of endothelial cells, thereby mimicking activated leucocytes. *P < 0.05.

To determine the feasibility of molecular MRI using targeted M3P in vivo, we used an experimental model of neuroinflammation in mice, induced by intrastriatal injection of *Escherichia coli* lipopolysaccharide (LPS). Twenty-four hours after injection of LPS (1.0 μg), brain MRI was performed both before and 3 min after iterative injections of M3P@αVCAM-1 corresponding to doses from 1.33 to 4 mg/kg of iron (Fig. 4, A and B, and movie S3). On precontrast images, no significant difference between the right and left hemispheres was observed in these mice on neither T2- nor T2*-weighted images. After intravenous injection of M3P@αVCAM-1, numerous signal voids were observed predominantly in the right striatum (Fig. 4A). Iterative injection demonstrated that higher doses led to more signal voids. The contralateral signal voids were expected in this model since it has already demonstrated that ipsilateral injection of pro-inflammatory cytokines in the striatum also induces contralateral inflammation, albeit at a milder level (*22*). We selected the dose of 4 mg/kg for further experiments to achieve high sensitivity while keeping within clinically tolerated doses of iron. Notably, M3P@αVCAM-1 was detectable at clinically relevant spatial resolution in this model and at this dose (fig. S27).

Thereafter, to investigate whether the signal voids of M3P@αVCAM-1 correlate with the severity of neuroinflammation, we administered different doses of LPS (0, 0.25, 0.5, or 1.0 μg) in the right striatum of naive mice. Twenty-four hours thereafter, we injected M3P@αVCAM-1 (4 mg/kg) intravenously and performed postcontrast T2*-weighted MRI of the brain. Consistent with a higher expression of VCAM-1, more signal voids were observed in the right hemisphere of the mice that received the highest doses of LPS (Fig. 4, C and D). Almost no signal void was observed in the brain of control mice, in line with a low basal expression of VCAM-1. Moreover, by comparing post- to preinjection images, we were able to generate three-dimensional (3D) maps of VCAM-1 expression in the brain in vivo and at high spatial resolution (movie S4).

### M3P@αVCAM-1 combines high sensitivity with high specificity

To investigate the specificity of our method, we compared M3P@αVCAM-1 to control M3P@IgG. Whereas M3P@αVCAM-1 induced numerous signal voids in the right striatum 24 hours after intrastriatal injection of LPS (1 μg), no signal void was visible in mice that received M3P@IgG (Fig. 4, E to G). Histological analyses confirmed the MRI findings by revealing significantly more M3P@αVCAM-1 than M3P@IgG in the right hemisphere of LPS-treated mice (fig. S28). By varying the ratio of IgG and αVCAM-1 on the surface of particles (0, 50, or 100% αVCAM-1/IgG ratio), we observed that the higher the concentration of targeted antibodies, the higher the binding of M3P@αVCAM-1 in the inflamed striatum (fig. S29). Moreover, when the mice were pretreated with αVCAM-1 monoclonal antibodies to saturate M3P@αVCAM-1 binding sites, no significant binding of M3P@αVCAM-1 was observed, confirming the specificity of the contrast agent (fig. S30).

Using high temporal resolution imaging, we also investigated the kinetic of M3P@αVCAM-1 binding on activated endothelial cells in the LPS model. As shown on fig. S31, the targeted particles rapidly accumulate in the right striatum, with near-maximal contrast reached less than 60 s after intravenous injection (movie S5). Control M3P@IgG only induced a short transient drop of T2*-weighted signal in the right striatum, consistent with a free flow through the microcirculation and a lack of binding. Both particles were rapidly cleared from the circulation with an estimated primary half-life of 50 to 70 s (fig. S31). At later time points (up to 14 days), T2*-weighted imaging revealed that progressive unbinding of the particles from the right striatum with near-complete disappearance of M3P@αVCAM-1 induced signal voids 24 hours after injection (fig. S32). Histological analyses revealed that unbound M3P@IgG accumulated macrophages in the liver (fig. S33). We also wanted to investigate whether the particles remained stable after incubation in whole blood (fig. S34). After 5 min of incubation, we did not detect large aggregates on blood smears from citrated whole blood, supporting that M3P@IgG is stable enough to remain dispersed during their circulating time after intravenous injection (5 min is equal to about five plasmatic half-lives of M3P). In contrast, we observed aggregates after 60 min of incubation. Considering their short plasmatic half-lives, these results support that conjugated M3P are stable enough in whole blood to avoid the formation of large aggregates before reaching their endothelial target. Together, these experiments demonstrate that M3P@αVCAM-1 is both sensitive and specific to reveal VCAM-1 overexpression in the brain vasculature.

### M3P improves the sensitivity of molecular imaging compared to classically used USPIO

**Fig. 5.**
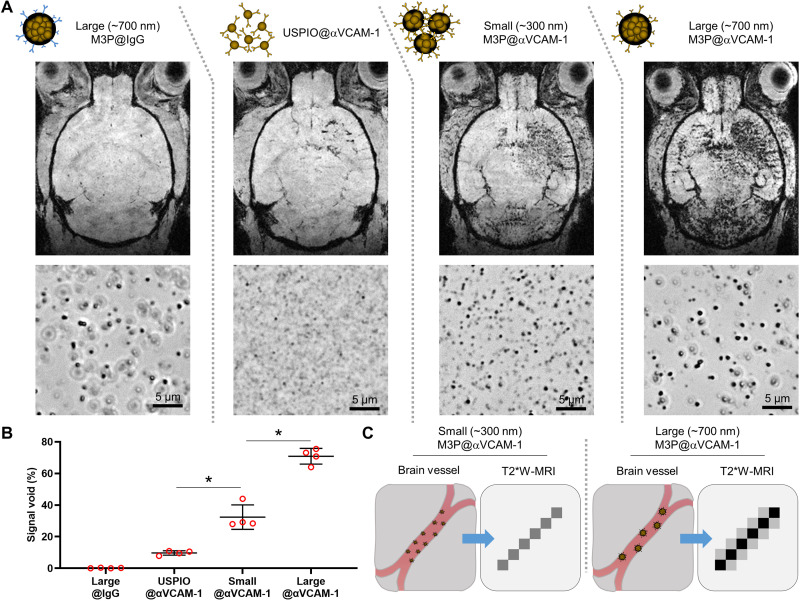
Clustering M3P into submicrometric particles improves sensitivity of molecular MRI of VCAM-1. (**A**) Top: Schematic representation of the particles. Middle: Representative T2*-weighted images after intravenous injection of the corresponding particles (4 mg/kg) 24 hours after injection of 1 μg of LPS in the right striatum. Bottom: Representative phase microscopy images of the particles after conjugation to control IgG or anti–VCAM-1 antibodies. (**B**) Corresponding quantification (*n* = 4 per group). (**C**) Schematic representation of the findings that large M3P (700 nm) provides better sensitivity than small M3P (300 nm). *P < 0.05.

To illustrate the gain in sensitivity provided by clustering superparamagnetic nanocrystals into microsized particles, we compared the sensitivity of unclustered USPIO and small (300 nm) and large (700 nm) M3P conjugated to anti–VCAM-1 monoclonal antibodies to reveal endothelial activation in vivo (Fig. 5A). In the LPS model of neuroinflammation, mice received 4 mg/kg of either particle. MRI was performed 20 min thereafter to allow clearance of the smallest particles. Quantitative analysis revealed significantly more signal void in the mice that received the largest particles (Fig. 5B). Almost no signal void was observed in mice that received control large M3P@IgG. These results support the improved sensitivity of large microsized particles compared to unclustered USPIO for molecular imaging of endothelial targets (Fig. 5C).

### M3P conjugated to antibodies targeting activated endothelial cells reveal inflammation in clinically relevant experimental models

**Fig. 6.**
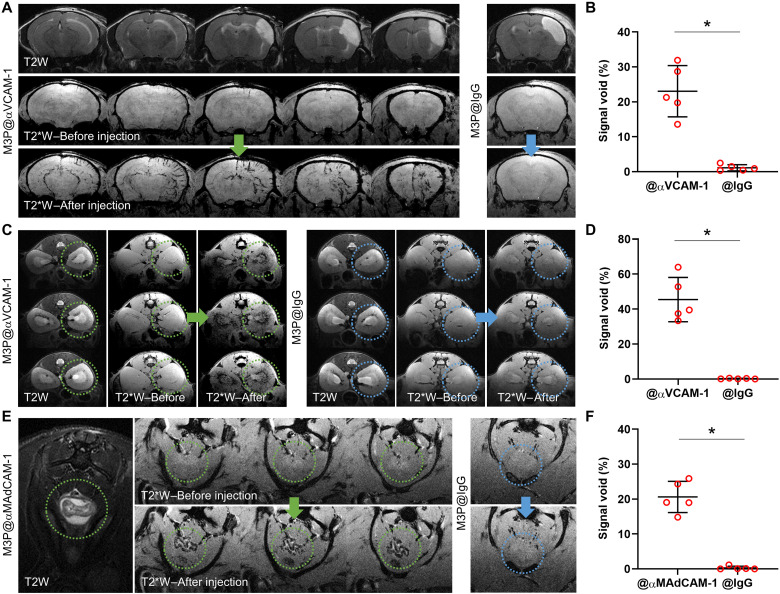
Molecular imaging of endothelial activation in clinically relevant experimental models. (**A**) Representative T2-weighted and T2*-weighted images before and after intravenous injection of M3P@αVCAM-1 (left) or M3P@IgG (right) (4 mg/kg) 24 hours after ischemic stroke induction by electrocoagulation of the right middle cerebral artery. (**B**) Corresponding quantification of signal void in the right hemisphere (*n* = 5 per group). (**C**) Representative T2-weighted and T2*-weighted images before and after intravenous injection of M3P@αVCAM-1 (left) or M3P@IgG (right) (4 mg/kg) 48 hours after acute kidney injury (rhabdomyolysis) induced by intramuscular injection of 50% glycerol. Yellow and blue dotted circles indicate the localization of the right kidney. (**D**) Corresponding quantification of signal void in the kidney medulla (*n* = 5 per group). (**E**) Representative T2-weighted and T2*-weighted images before and after intravenous injection of M3P@αMAdCAM-1 (left) or M3P@IgG (right) (4 mg/kg) in an acute colitis model induced by 5-day treatment with 2.0% of dextran sodium sulfate in the drinking water. Yellow and blue dotted circles indicate the localization of the descending colon. (**F**) Corresponding quantification of signal void in the descending colon (*n* = 5 per group). *P < 0.05.

Then, we performed molecular imaging of endothelial activation in more clinically relevant experimental models. The first is in a model of ischemic stroke induced by permanent middle cerebral artery occlusion (pMCAo). In this model, aseptic inflammation develops in the subacute phase (from 24 hours to 7 days after pMCAo), which is thought to play a key role in stroke pathophysiology (*23*). At 24 hours after pMCAo, intravenous injection of M3P@αVCAM-1 induced numerous signal voids in the right hemisphere in the periphery of the ischemic lesion (Fig. 6, A and B). In contrast, no significant signal void was observed after injection of control M3P@IgG. These data demonstrate that M3P@αVCAM-1 can unmask the neuroinflammatory reaction taking place in the subacute phase of ischemic stroke.

The second is in a model of acute kidney injury induced by rhabdomyolysis. An intramuscular injection of glycerol is performed in the two limbs to induce rhabdomyolysis, thereby releasing myoglobin from the muscles into the bloodstream and leading to subsequent acute kidney injury related to hypovolemia and direct toxicity of myoglobin on renal tubules. M3P@αVCAM-1 revealed endothelial inflammation mainly in the kidney medulla, in line with anatomical repartition of renal tubules (Fig. 6, C and D). Again, M3P@IgG did not induce any significant signal void.

The last is in a model of inflammatory bowel disease. Mice were treated for 5 days with dextran sulfate sodium (DSS) in the drinking water. DSS induces intestinal inflammation by disrupting the intestinal epithelial monolayer lining, leading to the entry of luminal bacteria and associated antigens into the mucosa, triggering an immune response. We performed molecular imaging of the descending colon both before and after injection of M3P@αMAdCAM-1, targeted to an adhesion molecule overexpressed by activated endothelial cells in mucosal tissues. As shown on Fig. 6 (E and F), intravenous injection of M3P@αMAdCAM-1 induced numerous signal void in the inflamed mucosa of DSS-treated mice, whereas M3P@IgG did not.

Together, these results demonstrate that immuno-MRI using targeted M3P can reveal inflammation in clinically relevant experimental models, as shown in three different organs (brain, kidney, and intestines) and with two different targets (VCAM-1 and MAdCAM-1).

## DISCUSSION

In the present study, we synthetized M3P, a new contrast agent for molecular MRI made of MNc embedded in a PDA matrix. The synthesis is based on the finding that MNc@Dopamine self-assemble into microsized particles in the presence of ammonia. M3P size can be tuned from 300 to 700 nm by varying the concentration of ammonia during synthesis. We demonstrated that these particles are water dispersible, biocompatible, and biodegradable and display favorable superparamagnetic properties. These M3P can be targeted through complexation to monoclonal antibodies (and theoretically to any molecules with free amine or thiol groups), representing a promising tool for immuno-MRI. In particular, we demonstrated that M3P coated with anti–VCAM-1 and anti–MAdCAM-1 antibodies are highly sensitive and specific to reveal inflammation by MRI in clinically relevant experimental models (summarized in fig. S35). We also demonstrated that this strategy is effective to target human activated endothelial cells, paving the way for clinical translation.

The use of PDA as a matrix presents several key features for molecular imaging. First, as a major component of naturally occurring neuromelanin, PDA is biocompatible and biodegradable in vivo, although the precise pathways for its biodegradation remain unknown (*24*). Second, a variety of ligand can be conjugated at high density with PDA via Michael addition or Schiff base reactions (*25*). The ability to conjugate a large amount of targeting moieties on the surface of M3P is required to maximize binding to the target and reach a high sensitivity (as illustrated in fig. S27). We cannot exclude however that a fraction of the antibodies on the M3P surface might be absorbed rather than covalently linked. Third, PDA is hydrophilic and negatively charged at physiological pH, providing a negative zeta potential for the coated particles and preventing their aggregation in solution. Last, dopamine interacts strongly with the surface of MNc through the phenolic hydroxyl groups (*26*). Thus, the PDA matrix in M3P is relatively resistant to ultrasonication (*27*), allowing dispersing aggregated particles without fragmenting the particles or detaching the conjugated ligands.

An important finding is the significantly improved sensitivity of molecular MRI using large particles compared to smaller ones. This result challenges the current focus of molecular imaging studies on nanosized contrast agents (<100 nm) (*28*). Considering that the shape of the particles remains the same, the amount of iron carried by a single particle increases proportionally to its radius at the power of 3. In our study, since the diameter of large M3P (700 nm) is 35 times larger than classically used USPIO (20 nm), the amount of contrast material carried per particle is >40,000 times larger. Even if it is conceivable that smaller particles can accumulate more efficiently on the endothelial surface thanks to a longer circulation time, our data show that the use of large M3P is much more effective. Previous studies also reported an improved margination on the vessel wall of large particles compared to smaller ones (*29*) and better performances of large particles for molecular MRI of endothelial targets (*30*). These results support the use of micrometric or submicrometric particles rather than nanoparticles for molecular imaging of endothelial activation, especially when the sensitivity of the imaging modality is limited. Understandably, there is an upper limit of the size of rigid particles to allow free circulation through capillary vessels. Notably, the use of nanosized crystallites inside the M3P remains mandatory to preserve the superparamagnetic behavior and avoid particle aggregation.

Beyond improved sensitivity, this larger size also prevents passive extravasation and accumulation of the contrast carrying particles and, therefore, improves the specificity of the signal. This comes at the cost of a strictly intravascular biodistribution, limiting imaging to vascular targets, and a short half-life due to rapid uptake by the reticuloendothelial system (*13*, *31*). Accordingly, the plasmatic half-life of targeted 700-nm M3P is very short, with near-complete plasma clearance achieved a couple of minutes after intravenous injection. This is similar to what has been observed previously using microsized magnetic particles both in terms of circulation half-life and biodistribution pattern (*30*). This pharmacokinetic profile allows performing postcontrast MRI almost immediately after intravenous injection, which would be useful in clinical practice to ease patient workflow.

Although the increase in sensitivity to detect endothelial activation allowed by large contrast-carrying particles is valuable to image the immune response, it has also some limitations. First, some level of endothelial activation and adhesion molecule expression can be observed in quiescent tissues, albeit at much lower intensity than in inflamed tissues. Therefore, it is important to consider the degree of signal changes after M3P injection to accurately identify inflammatory versus quiescent tissue. It is also important to acknowledge that molecular imaging of endothelial activation using targeted M3P gives only a semiquantitative information on the extent of inflammation. Second, in the context of focal injury, the inflammatory reaction can be so intense that highly sensitive imaging can detect endothelial activation far from the injury site (*32*). In such conditions, targeted M3P-enhanced imaging will lack spatial selectivity to precisely identify the site of injury. Moreover, because of the constraints inherent in clinical imaging, including lower magnetic field, lower spatial resolution, and possibly lower iron dose, it is expected that the sensitivity of M3P-enhanced imaging will be lower in humans. It is however reinsuring that M3P@αVCAM-1 is easily detected at clinically relevant spatial resolution at 24 hours after intrastriatal LPS injection in mice.

Overall, M3P present favorable characteristics for clinical translation. The synthesis is easy to perform at room temperature and atmospheric pressure and should be amenable to large-scale production, and the required reagents are cheap and widely available. The sensitivity for molecular MRI of endothelial activation is high enough to detect inflammation in clinically relevant model and at clinically relevant spatial resolution. The iron dose used in the present study (4 mg/kg) was slightly higher than the dose reported in clinical studies that used nanosized particles of iron oxide for neuroimaging (2.6 mg/kg) (*33*). Notably, in the context of the treatment of iron deficiency, much higher doses of USPIO (up to 1.5 g in a single injection) are well tolerated in humans (*34*). Since MRI is a routinely performed imaging technique and our contrast agent can be detected using standard sequences and magnets, M3P could be rapidly and widely adopted in clinical practice. A minor modification of currently performed imaging protocols could allow the inclusion of a postcontrast T2*-weighted sequence, therefore allowing M3P detection. To enable clinical translation, several potential limitations of M3P remain to be addressed including evaluation of the risk of anaphylaxis that has been reported for iron oxide nanoparticles, the putative short- and long-term effects of rapid liver and spleen accumulation after intravenous injection, the risk of particle aggregation once in the blood and subsequent small vessel embolization, and the risk of immunological reactions. The risks associated with intravenous injection of micrometric and submicrometric particles also remain unknown since, to our knowledge, there are no clinically approved biomaterials in this size range for intravenous use. Among the most straightforward applications of this technology are early diagnosis of autoimmune diseases, prediction of treatment response to immune checkpoint inhibitors in cancer, and noninvasive monitoring of immunomodulatory therapies. While additional translational hurdles will need to be considered for future clinical development, M3P provides a promising platform for the clinical translation of immuno-MRI.

## MATERIALS AND METHODS

### Study design

The main objective of this study was to synthesize a microsized magnetic particle that is fully biodegradable and to demonstrate the feasibility of molecular imaging of inflammation in vivo using this particle. We determined sample sizes in the in vivo MRI experiments based on our previous experience. We commonly used four to five mice per group to measure the signal changes induced by contrast agent administration compared to control condition (mice with inflammation versus mice without inflammation, targeted M3P versus control M3P, etc.). This sample size was large enough to detect differences in these all-or-nothing experimental paradigms. No animal was excluded from the analysis. All animals were randomized to the different experimental groups, ensuring equivalent representation of each animal cage in each experimental group. Twenty-three batches of M3P were produced for this study, and consistent results were obtained with all batches. All the animals from the same experiments received M3P from the same batches to avoid putative batch differences. All image analyses were performed blinded to the experimental data using the anonymization procedure of the MRI software.

### Reagents for M3P production

The following reagents were purchased from Sigma-Aldrich: ferric chloride hexahydrate, ferrous chloride tetrahydrate, ammonia solution (32%), dopamine hydrochloride, sodium phosphate monobasic, sodium phosphate dibasic, and mannitol. Antibodies for conjugation to M3P were rat anti-mouse VCAM-1 [clone A(429), BD Biosciences], mouse anti-human VCAM-1 (clone 1.G11B1, Thermo Fisher Scientific), and rat anti-mouse MAdCAM-1 (clone MECA367, BD Biosciences), and corresponding isotype polyclonal IgG were purchased from Sigma-Aldrich.

### Synthesis of MNcs

MNc were produced by a coprecipitation method in an alkaline buffer. In a typical synthesis, 540 mg of FeCl_3_.6H_2_O and 198.8 mg of FeCl_2_.4H_2_O were dissolved in 5.7 ml of distilled water by vortexing, yielding a homogenous yellow solution. Under continuous agitation at room temperature, 6.3 ml of a 13% ammonia solution was progressively added at a rate of 0.2 ml/min. The solution turned from yellow to brown and, ultimately, to a deep black color, corresponding to the formation of magnetite. The precipitate was washed five times with distilled water by magnetic separation and resuspended in 10 ml of distilled water.

### Synthesis of dopamine-coated MNcs

Eight milliliters of the solution of MNc was resuspended in 40 ml of a solution containing dopamine hydrochloride (2.5 mg/ml). The resulting solution was sonicated for 15 min at 70% amplitude and 26 kHz using a UP200ST sonicator (Hielscher). The color of the solution slightly changed from black to dark brown. Then, the solution was centrifuged at 3000*g* for 5 min to remove large remaining nanocrystals aggregates. Thirty milliliters of the supernatant containing MNc@Dopamine and free dopamine hydrochloride were transferred to a new vial.

### Synthesis of M3P

The solution containing MNc@Dopamine and free dopamine hydrochloride was placed under vigorous steering using an Ultra-Turrax T-25 disperser at 20,500 rpm. To produce large M3P (700 nm), 67 μl of a 13% ammonia solution was first added to the solution, which was left to react for 60 min. Then, 203 μl of a 13% ammonia solution was added, and the incubation was continued for 30 min to allow further PDA. To produce medium-sized M3P (500 nm), 270 μl of a 13% ammonia solution was added in one time and the solution was left to react for 60 min. To produce small-sized M3P (300 nm), 2160 μl of a 13% ammonia solution was added in one time and the solution was left to react for 60 min. Then, the solution was centrifuged at 1000*g* for 3 min to remove the largest aggregates, the pellet was discarded, and 24 ml of the supernatant was transferred to a new vial. The M3P were then washed five times with distilled water and lastly resuspended in 8 ml of distilled water and stored at 4°C until further use.

### Determination of iron concentration

Iron content of M3P suspension was measured with the FerroZine method. Particles were degraded overnight at room temperature in 1 M HCl, releasing ferric (Fe^3+^) and ferrous (Fe^2+^) ions in solution. Samples were incubated 30 min with 0.65% (w/v) ascorbic acid to reduce ferric ions in ferrous ions. Sample pH was adjusted with 12% (w/v) ammonium acetate. 3-(2-Pyridyl)-5,6-diphenyl-1,2,4-triazine-*p*,*p*′-disulfonic acid monosodium salt hydrate was added (1 mM; FerroZine Iron Reagent, Sigma-Aldrich), and absorbance was measured at 562 nm with a spectrophotometer (ELx808 Absorbance reader, BioTek) indicating the amount of complexes formed with ferrous ions. Iron content was determined against standard curves obtained from iron chloride dilutions.

### Determination of the hydrodynamic diameter of the particles

Dynamic light scattering was used to determine the average hydrodynamic diameter, the polydispersity index and the diameter distribution by volume of the M3P particles with a Nano ZS apparatus (Malvern Instruments, Worcestershire, UK) equipped with a 633-nm laser at a fixed scattering angle of 173°. The temperature of the cell was kept constant at 25°C, and all dilutions were performed in pure water. Measurements were performed in triplicate.

### Zeta potential measurement

Zeta potential analyses were realized, after 1/100 dilution in 1 mM NaCl, using a Nano ZS apparatus equipped with DTS 1070 cell. All measurements were performed in triplicate at 25°C, with a dielectric constant of 78.5, a refractive index of 1.33, a viscosity of 0.8872 centipoise, and a cell voltage of 150 V. The zeta potential was calculated from the electrophoretic mobility using the Smoluchowski equation.

### Thermogravimetric analysis

TGA was achieved on a TA SDT 600 instrument to measure the mass fractions of the organic and inorganic parts of the M3P. For the runs, the samples were heated from 25° to 800°C at a rate of 5°C/min under air.

### X-ray diffraction

The x-ray diffractograms of the M3P were obtained at ambient temperature with a Bruker D8 Advance diffractometer (Bruker, Billerica, MA, USA) equipped with a monochromatic copper radiation source (Kα = 0.154 056 nm) and a LYNXEYE detector in the 20° to 70° (2θ) range with a scan step of 0.03°. High-purity silicon powder (*a* = 0.543 082 nm) was used as an internal standard. Profile fitting refinements were realized via the FullProf program using Le Bail’s method with the modified Thompson-Cox-Hasting pseudo-Voigt profile model.

### Superconducting quantum interference device

Hysteresis cycles at 5 K and 300 K of the M3P were performed with a superconducting quantum interference device (Quantum Design MPMS3). For that, 50 μl of M3P suspensions at ca. 7 mg of Fe/ml was dried and evaporated in a capsule. This procedure was done again until the dried M3P cover the capsule bottom part.

### Measurements of the relaxivities by ^1^H nuclear magnetic resonance spectroscopy

The longitudinal T1 and transverse T2 relaxation times of the samples were measured by using a Bruker Minispec 60 (Karlsruhe, Germany) apparatus working at 37°C, at a field of 1.41 T corresponding to a Larmor frequency of 60 MHz. The corresponding longitudinal (R1) and transverse (R2) relaxivities were calculated according to the following expression involving the contrast agent: *R* = R0 + *r* × [Fe], where [Fe] is the iron concentration of the samples, *R* is the relaxation rate (1/*T*) (in the presence of the particles), R0 is the relaxation rate of the aqueous solution (in the absence of the particles), and *r* is the fitted relaxivity value.

### Measurements of the relaxivities by 1H 7-T MRI

Large 700-nm M3P was dispersed in agarose gels (2%) in tris-acetate-EDTA buffer at different concentrations. The samples were then imaged using a BioSpec 7-T TEP-MRI, and the following sequences were performed: T1 mapping using flow-sensitive alternating inversion recovery–rapid acquisition with relaxation enhancement (RARE) sequence with repetition time (TR) = 3000 ms and inversion time ranging from 6.5 to 2000 ms, T2 mapping using multislice multiecho (MSME) sequence with TR = 4000 ms and echo time (TE) ranging from 3.65 to 51.11 ms, and T2* mapping using multigradient echo sequence with TR = 4000 ms and TE ranging from 2 to 17.47 ms. The corresponding R1, R2, and R2* relaxivities were calculated as described above.

### In vitro degradation in buffers

Stability and biodegradability of small, medium, and large M3P, as well as MNc@Dopamine, were studied in vitro in different relevant biological buffer solutions: ALF, acidic solution [20 mM citrate buffer (pH 4.0)], and citrate + H_2_O_2_ [20 mM citrate buffer (pH 4.0) + 30 mM H_2_O_2_] buffer. PBS was used as a control buffer. ALF was prepared as previously reported by Milosevic *et al*. (*35*) Briefly, sodium chloride (3.210 g), sodium chloride (0.097 g), sodium hydroxide (6.000 g), citric acid (20.800 g), calcium chloride (0.097 g), sodium phosphate heptahydrate (0.179 g), sodium sulfate (0.039 g), magnesium chloride hexahydrate (0.106 g), glycerin (0.059 g), sodium citrate dehydrate (0.077 g), sodium tartrate dehydrate (0.090 g), sodium lactate (0.085 g), sodium pyruvate (0.086 g), and formaldehyde (1000 ml added fresh before use) were dissolved in 200 ml of purified water to obtain a 5× stock solution, which was later diluted with purified water and particles during incubation. To assess particle stability and/or dissolution in PBS, ALF, citric, and citrate + H_2_O_2_ buffers, all four particles types were incubated in those buffers [at Fe (20 μg/ml)], and consequently, UV-vis spectra were measured and images of the dispersion were taken at the beginning (baseline), 3 hours, 24 hours, 72 hours, and 7 days (the end of the incubation).

### In vitro degradation in THP-1 macrophages

THP-1 cells (American Type Culture Collection) were cultivated in RPMI 1640 medium (Sigma-Aldrich) supplemented with 10% fetal calf serum and 2% penicillin-streptomycin, at 37°C with 5% CO_2_. Cells were plated in a 24-well plate over glass microslides at 500,000 cells per well and differentiated into macrophages with phorbol 12-myristate 13-acetate (2 μM, Sigma-Aldrich). At 24 hours, cells in suspension were removed and 700-nm M3P or nondegradable MPIO (Dynabeads MyOne Carboxylic Acid, Thermo Fisher Scientific) were added at a final iron content of 15 μg/ml. At 96 hours, THP-1 macrophages were processed for transmission electron microscopy (TEM).

### Transmission electron microscopy

For observation of M3P or MPIO in suspension, a droplet of particles was after deposited on a hydrophilized 400-mesh grid. High-resolution TEM was performed on an aberration-corrected JEOL ARM 200-F microscope operating at 80 kV (Laboratoire Matériaux et Phénomènes Quantiques, UMR7162, Université de Paris/CNRS, France). For observation of THP-1 cells, the cells were cultured and incubated with particles on microscope coverslips. Cells were fixed with 2.5% glutaraldehyde in 0.1 M cacodylate buffer (pH 7.4) overnight at 4°C, rinsed in 0.1 M cacodylate buffer (pH 7.4), postfixed 1 hour with 1% osmium tetroxide in 0.1 M cacodylate buffer (pH 7.4) at 4°C, and rinsed in 0.1 M cacodylate buffer (pH 7.4). The cells were then dehydrated in progressive bath of ethanol (70 to 100%) and embedded in resin EMbed 812. After 20 hours of polymerization at 60°C, the coverslip were then separated from the cell’s resin bloc and polymerization continued for 28 hours. Ultrathin sections were collected and contrasted with uranyl acetate and lead citrate. Sections were observed with TEM JEOL 1011, and images were taken with Camera MegaView 3 and AnalySIS FIVE software.

### Clot lysis

The effect of M3P during clot formation and lysis was studied by monitoring the change in turbidity in human or mouse plasma using a microplate reader (FLUOstar Optima, BMG Labtech). Calcium chloride (final concentration, 25 mM) was added to citrated plasma diluted 1:2 in Hepes buffer [10 mM Hepes, 150 mM NaCl, and 0.4% bovine serum albumin (BSA) (pH 7.4)] to promote coagulation. Samples were incubated at 37°C with M3P at different doses, and absorbance (405 nm) was monitored for 12 hours every 30 s at 37°C. When appropriate, recombinant tissue-type plasminogen activator (alteplase, Boehringer Ingelheim) was also added to promote clot lysis. Results are expressed as the time to achieve 75% maximal absorbance [clotting time (CT)], and the 50% lysis time was calculated as the time from initiation of clot formation to the time at which maximal absorbance falls to 50%. All experiments were performed in triplicate.

### Rotational thromboelastography

For thromboelastography assays, human fresh blood was drawn into 0.109 M trisodium citrate. Within 30 min, 300 μl of blood was added in EXTEM-S reagent vial (EXTEM) in the presence of increasing concentrations of large M3P [0 (control), 20, 40, and 80 μg/ml] diluted in saline and clotting was monitored. CT (in seconds), amplitude at 10 min (A10), clot formation time (CFT; in seconds), maximal clot firmness (in millimeters), and α angle (in degrees) were measured to characterize and quantify the coagulation activation, clot firmness, and clot stability using rotational thromboelastography (TEM). Blood of healthy donors (Etablissement Français du Sang, Ile de France) was collected and tested with the appropriate ethics prior approval as stated in the EFS/INSERM U1237 agreement #PLER-UPR/2018/017, ensuring that all donors gave a written informed consent and providing anonymized samples.

### Erythrocyte hemolysis assay

The erythrocyte toxicity of the M3P was conducted as described by Mazzarino *et al*. (*36*) with minor modifications on human and mouse blood. Briefly, 1 ml of blood was obtained by venous (human) or intracardial puncture (mice) in 1/10 citrate buffer. Erythrocytes were purified by centrifugation at 2000*g* for 5 min and washed three times in 10 ml of saline. Then, 50 μl of purified and washed erythrocytes was added to 950 μl of saline containing different concentrations of M3P (final concentration, 0, 20, 40, and 80 μg/ml) and incubated in 37°C under mild agitation. Then, erythrocytes were removed by centrifugation at 2000*g* for 5 min. Then, the absorbance of the resulting supernatant was measured by UV-vis and by spectrometer at 540 nm. Saline control solution (0.9% NaCl) and distilled water were used as negative (0% lysis) and positive (100% lysis) controls, respectively. The hemolysis rate at 540 nm was calculated as follows: hemolysis rate (%) = (*A*_s_ – *A*_c−_/*A*_c+_ − *A*_c−_) × 100, where *A*_s_, *A*_c−_, and *A*_c+_ represent the absorbance of the sample, negative control, and positive control, respectively (*37*). The experiments were performed in triplicate using either human or mouse erythrocytes.

### In vitro cell death screening studies

In vitro studies using primary cultures of HUVECs (Merck KGaA, C-12203) were used to assess acute and chronic toxicity of M3P. To allow for the more efficient screening, we combined two independent tests into one cell death screening assay developed by Filipova *et al*. (*38*) with minor modifications: first, the cell proliferation and viability test based on WST-1 (4-[3-(4-iodophenyl)-2-(4-nitro-phenyl)-2H-5-tetrazolium]-1,3-benzene sulfonate, monosodium salt) assay (Roche) and, second, the cell membrane integrity and cell necrosis by lactate dehydrogenase (LDH; Cytotoxic Detection Kit, Roche) assay. Both assays were performed in sequence on the cells in the same well. Briefly, HUVECs were obtained from Merck and were cultured in precoated 0.2% gelatin in 25-cm^2^ flasks in endothelial cell growth medium (EGM-2; Lonza) containing 2% fetal bovine serum (FBS) and supplements (Lonza BulletKit, CC-3202) at 37°C in a humidified 5% CO_2_ atmosphere. After reaching ~85% confluence, cells were harvested in trypsin/EDTA phenol red for endothelial cells (Gibco) and centrifuged at 300*g* for 5 min, and 10 × 10^3^ cells were seeded in 200 μl of fresh EGM-2 media per well in 96-well plates (Thermo Fisher Scientific). After 48 hours, HUVECs were treated in five replicates with increasing concentrations of large M3P (in saline solution) from 0 to 320 μg/ml diluted in saline solution (dose-response), positive control (1% Triton X-100), and negative control (freshly added medium). Plate was incubated for 0, 3, and 24 hours (time-response experiment). Then, 50 μl of supernatants was transferred into a new 96-well plate. The LDH assay was performed following the manufacturer’s instructions by measuring absorbance at 490 and 660 nm. Thereafter, same wells containing 150 μl of media were treated with 15 μl of WST-1 reagent and were incubated for 1 hour at 37°C. Subsequently, 100 μl of the media with developed color was transferred to a new 96-well plate, and absorbance was measured at 450 and 660 nm. The net absorbance corresponding to the LDH and WST-1 (after elimination of M3P contribution to absorbance) was calculated following the corrective formulas of Filipova *et al*. (*38*). Both values of cell viability and integrity are expressed as a percentage of the negative control values. Besides, impact of cell viability after M3P addition at increasing doses was performed by a follow-up of histological images. In this experiment, 3 × 10^6^ HUVEC cells were seeded in 2 ml of fresh EGM-2 media per well in six-well plates (Thermo Fisher Scientific). After 48 hours, HUVECs were treated in two replicates with increasing concentrations of large M3P from 0, 80, 160, to 320 μg/ml diluted in purified water. Images were taken at baseline, 20 min, 3 hours, 6 hours, and 24 hours (before and after washing). Negative control was freshly added medium. For all experiments, cells were used at third to fourth passage.

### Optimization of antibodies binding to M3P surface

To calculate the best concentration of antibodies at the surface of M3P, we performed a dose-response experiment using rat IgG. To that aim, 560 μg of large M3P was washed one time with purified water and resuspended in 1 ml of 10 mM phosphate buffer (pH 8.5). Then, dose escalation of 0 to 225 μg of rat IgG (0, 45, 90, 135, 180, and 225 μg) was incubated with M3P at room temperature for 24 hours. In parallel, the resulting solutions were washed in 1/300 ammonia solution (from a 13% ammonia solution) and sonicated for 1 min at 20% amplitude and 26 kHz using a UP200ST sonicator to break any aggregates. The supernatants were kept to measure the concentration of unbound antibodies by UV-vis and Coomassie SDS-PAGE. In parallel, the coated M3P were then washed twice with a 0.3 M mannitol solution and incubated with secondary anti-rat fluorescein isothiocyanate (FITC) secondary antibodies (or anti-rabbit FITC for isotype mimicking control condition in borate buffer for 30 min at room temperature in the dark). After incubation, M3P solutions were washed twice and resuspended in PBS and 2% BSA buffer for acquisition in the flow cytometer. M3P were acquired on a FACSVerse cytometer (BD Biosciences). The results were analyzed using FlowJo version 7.6.5 (BD Biosciences).

### Coating of M3P with antibodies

In a typical coating procedure, 2.5 mg of M3P was washed one time with purified water and resuspended in 5 ml of 10 mM phosphate buffer (pH 8.5). Then, 400 μg of monoclonal antibodies (or another appropriate amount) was incubated with M3P at room temperature for 24 hours. The resulting solution was sonicated for 5 min at 20% amplitude and 26 kHz using a UP200ST sonicator to break any aggregates. The coated M3P were then washed three times with a 0.3 M mannitol solution and lastly resuspended in 5 ml of a 0.3 M mannitol solution and stored at 4°C until further use.

### Production of USPIO@αVCAM-1

We first produced MNc@Dopamine as described above. Then, the excess dopamine was removed by dialysis against purified water (10-kDa cutoff). Thereafter, the purified nanoparticles were incubated with anti–VCAM-1 antibodies in a phosphate buffer [10 mM (pH 8.5)] to obtain USPIO@αVCAM-1 by direct bioconjugation (using the same antibody/iron ratio as during the conjugation of M3P, i.e., 160 μg/mg). The excess antibody was removed by several cycles of magnetic purification, and last, USPIO@αVCAM-1 was resuspended in an isotonic mannitol buffer and briefly sonicated to break any aggregates. Using this methodology, USPIO@αVCAM-1 and the two M3P@αVCAM-1 formulation (small and large) were made of the same nanocrystals of iron oxide, were conjugated using the same antibody/iron ratio, and were injected at the same dose (4 mg/kg), thereby allowing to investigate specifically the effect of particle size on imaging sensitivity.

### In vitro binding of M3P@αVCAM-1 to TNF-activated human brain endothelial cells

Chamber experiments were performed to assess the binding affinity of anti–VCAM-1 antibody conjugated to M3P to human activated brain endothelial cells. To that aim, the hCMEC/D3 was cultured as described previously (*39*). Endothelial cells were cultured in precoated rat collagen-I 25-cm^2^ flasks in EGM-2 (Lonza) containing 2% FBS and supplements (Lonza BulletKit, CC-3202) at 37°C in a humidified 5% CO_2_ atmosphere. The media were renewed every 2 days until confluence was reached at ~85%, after which the cells were harvested in trypsin/EDTA phenol red for endothelial cells (Gibco), centrifuged at 300*g* for 5 min, and seeded in 300 μl of fresh EGM-2 media per well in Ibidi μ-Slide 8 well ibiTreat (#CC80826) at 5 × 10^4^ cells/ml. After 24 hours, hCMEC/D3 cells were changed to endothelial cell serum-free media (Gibco) and exposed to human TNF (50 ng/ml; activated condition; #300-01A, PeproTech) or PBS (for the control/quiescent condition) for >16 hours to induce endothelial VCAM-1 expression. After that, control and stimulated cells were washed with fresh EGM-2 media and incubated in duplicates with either M3P@αVCAM-1 or M3P@IgG (40 μg/ml) for 30 min at room temperature with constant mixing. Unbound particles were removed by extensive washing with PBS. Thereafter, cells were fixed with 4% paraformaldehyde (PFA) for 10 min, washed three times with PBS, and permeabilized with 0.1% Triton X-100-PBS solution for 15 min at room temperature. To visualize bound M3P (either VCAM-1 or IgG), anti-mouse specific secondary antibodies conjugated for Cy3 were purchased from Jackson ImmunoResearch Laboratories. To visualize the actin protein, iFluor488-phalloidin (#176753 Cytopainter, Abcam), a bicyclic heptapeptide that specifically binds to F-actin protein, was used. Slides were coverslipped with Fluoromount-G containing 4′,6-diamidino-2-phenylindole (DAPI) for nuclei staining (Thermo Fisher Scientific). Confocal images were acquired on an SP8 confocal microscope (40× objective; Leica Microsystems) and captured at 1024 × 1024 high-quality resolution with a *Z* step of 0.10 μm. The image data were analyzed, processed, and reconstructed with ImageJ 1.5 software (National Institutes of Health). M3P@αVCAM-1 and M3P@IgG binding to cells was assessed in five fields of view per TNF and PBS dose and quantified using ImageJ. To confirm the overexpression of VCAM-1 in brain endothelial cells after TNF stimulation, hCMEC/D3 was stimulated with TNF for 24 hours (or PBS for quiescent conditions), washed with PBS, and detached using trypsin-EDTA.Quiescent (PBS) and activated (TNF) cells were centrifuged (300*g* for 5 min) and resuspended at 1 × 10^5^ cells in 100 μl of PBS. Primary antibodies to anti-mouse VCAM-1 coupled to FITC (Invitrogen) and isotype controls were incubated in both groups in PBS + BSA 2% at a final concentration of 10 μg/ml for 30 min at room temperature under the dark. Cells were washed twice in PBS + BSA buffer. Flow cytometry experiments were performed on a FACSVerse flow cytometer (BD Biosciences) equipped with its accompanying software (FACSuite, BD Biosciences) on 20,000 events after gating the population by forward scatter and side scatter and performing FITC density plots.

### Animals

All experiments were performed on 8- to 16-week-old male Swiss mice (Janvier, France). Animals were maintained under specific pathogen–free conditions at the Centre Universitaire de Ressources Biologiques (CURB, Basse-Normandie, France), and all had free access to food and tap water. Experiments were approved by the local ethical committee of Normandy (CENOMEXA, APAFIS#22318). A catheter was inserted into the tail vein of mice for intravenous administration of the contrast agent.

### Inflammatory cytokines and transaminases dosage

Blood samples were collected from the inferior vena cava on either 3.2% (w/v) sodium citrate or heparinized tubes. Plasma was isolated by centrifugation (2× 1500*g* for 15 min) and kept at −20°C until use. Inflammatory cytokines were quantified in citrated plasma samples with a multiplex sandwich immunoassay using a pro-inflammatory panel kit for mouse (MSD multi-spot assay system, V-PLEX). Concentrations of TNF-α, interleukin-1β (IL-1β), IL-2, IL-6, interferon-γ, and CXCL1 were determined in picograms per milliliter with an electrochemiluminescent substrate by backfitting the signal to calibration curves with Discovery Workbench software (v 4.3). Alanine and aspartate aminotransferase activities were determined using an AU5800 chemistry analyzer (Beckman Coulter) on heparinized plasma with blank determination and quality controls (three different levels) performed as described by validated quality procedures. These assays are based on transamination of alanine in pyruvate and aspartate into oxaloacetate coupled to a second reaction where these alpha-keto acids are reduced in the presence of Nicotinamide adenine dinucleotide; the reaction was followed at 340 nm.

### Biodistribution study

Mice were anesthetized with isoflurane (1.5 to 2.0%) and maintained at 37°C, and M3P were injected intravenously (4 mg/kg). At 1 hour, 24 hours, 7 days, 1 month, and 6 months after injection, 0.5 to 1 ml of blood was collected from the inferior vena cava on 3.2% (w/v) sodium citrate, mice were perfused with saline, and tissues of interest were collected: liver, spleen, kidneys, lung, and heart. Plasma was isolated from blood samples by centrifugation (2× 1500*g* for 15 min) and kept at −20°C for pro-inflammatory cytokine and transaminase dosages. Tissues were weighted and divided for various analyses. A piece of liver, one kidney, half of the lung, half of the heart, and half of the spleen were weighted and frozen at −80°C for EPR analysis. The rest of the tissues were fixed in 4% phosphate-buffered PFA for histology studies. The study was designed with one group per time point with additional groups of mice treated for 24 hours with intraperitoneal injection of LPS (5 mg/kg) and mice injected with vehicle control (*n* = 5 mice per group).

### Electron paramagnetic resonance

EPR was performed with a Bruker ELEXSYS 500 EPR spectrometer operating at X band (9 GHz). The first derivative of the power absorption *dW*(*B*)/*dB* was recorded as a function of the applied field *B* in the range 0 to 6000 G, with a modulation field at a frequency of 100 kHz and amplitude of 10 G.

The area of the EPR absorption curve [calculated by a double integration of the spectrum *dW*(*B*)/*dB*] was proportional to the amount of nondegraded superparamagnetic M3P in the sample. An absolute calibration was performed using suspensions of initial M3P in 2-μl sample at different iron concentrations. For quantification of superparamagnetic components in ex vivo tissue, excised pieces of organs were first weighed and let dry for 3 days in an oven at 80°C. The dried organ samples were weighed again and introduced into a quartz tube. The net mass of dehydrated organs in the quartz tube was then weighed, and EPR spectra were recorded.

### Intrastriatal LPS injection

Swiss mice received an intrastriatal injection of LPS from *E. coli* (1.5 μg per mouse; 0111:B4; Sigma-Aldrich, L’isle d’Abeau, France) that was performed after placing male Swiss mice in a stereotaxic frame (coordinates: 0.5 mm anterior, 2.0 mm lateral, −3 mm ventral to the bregma) under isoflurane anesthesia (2.0%). Solutions were injected by the use of a glass micropipette.

### Permanent middle cerebral artery occlusion

Male Swiss mice were placed in a stereotaxic frame under isoflurane anesthesia (2.0%); the skin between the right ear and the right eye was incised, and the temporal muscle was carefully retracted. Then, the middle cerebral artery was exposed through a small craniotomy and permanently occluded by electrocoagulation.

### Rhabdomyolysis-induced acute kidney injury

To induce rhabdomyolysis, the animals were injected intramuscularly, on each thigh caudal muscle, with 50% glycerol (7.5 ml/kg) [99.5% (m/v); VWR International S.A.S., France] or saline as a control.

### DSS model of acute colitis

DSS-treated mice received 2.0% (w/v) DSS (40,000 molecular weight; Alfa Aesar, Thermo Fisher Scientific, France) in drinking water for 5 days to induce an acute episode of colitis. Drink and food were provided ad libitum. DSS, food consumption, and body weight were measured daily.

### Whole-body MRI

Experiments were performed using a BioSpec 7-T TEP-MRI system with a volume coil resonator (Bruker, Germany). Mice were anesthetized with isoflurane (1.5 to 2.0%) and maintained at 37°C by the integrated heat animal holder, and the breathing rate was monitored during the imaging procedure. Whole-body scans including T2-weighted (RARE sequence, with TR/TE = 3000 ms/50 ms) and T2*-weighted sequences [fast-low angle shot (FLASH) sequence, with TR/TE = 50 ms/3.5 ms] were performed before, 20 min, 24 hours, 7 days, 1 month, and 6 months after the intravenous injection of 700-nm M3P (4 mg/kg). Signal intensity ratios were measured by drawing the region of interest in the liver, spleen, kidney, and paravertebral muscles. Ratios were computed as the signal intensity of the organ of interest divided by the signal intensity of the paravertebral muscle (*n* = 7).

### In vivo immuno-MRI

Experiments were carried out on a Pharmascan 7-T/12-cm system using surface coils (Bruker, Germany). Mice were anesthetized with isoflurane (1.5 to 2.0%) and maintained at 37°C by the integrated heat animal holder, and the breathing rate was monitored during the imaging procedure. To image the brain, T2-weighted images were acquired using an MSME sequence (spatial resolution of 70 μm by 70 μm by 500 μm) with TE/TR 51 ms/2500 ms and a flip angle of 90°. T2*-weighted images were acquired using a 3D FLASH gradient echo imaging (spatial resolution of 78 μm by 78 μm by 150 μm) with TE/TR 8.6 ms/50 ms, and a flip angle of 20° was performed to reveal M3P and USPIO (acquisition time, 17 min). High-resolution T2*-weighted images presented in this study are minimum intensity projections of three consecutive slices (yielding a *Z* resolution of 450 μm). Fast acquisition of the brain for biodistribution study was performed using a FLASH sequence with only one slice and TR/TE 20 ms/7 ms with a flip angle of 8°. To image the kidneys, T2-weighted images were acquired using a turbo RARE sequence (spatial resolution of 78 μm by 78 μm by 500 μm) with TR/TE 2500 ms/40 ms and RARE factor of 8. T2*-weighted images were acquired using a FLASH sequence with TR/TE 250 ms/5 ms and a flip angle of 30°. To image the colon, T2*-weighted images were acquired using a 2D FLASH sequence (spatial resolution of 67 μm by 67 μm by 350 μm) without gating with TE/TR 5.4 ms/300 ms and a flip angle of 35°. T2-weighted images were acquired using a 2D RARE sequence (spatial resolution of 67 μm by 67 μm by 750 μm) with TE/TR 36 ms/2500 ms and a flip angle of 90°. When appropriate, mice randomly received M3P or appropriate control. When appropriate, T2*-weighted images were acquired both before and after injection of contrast agent. Although injections of contrast agent were usually performed outside the magnet, the positions of the mice during pre- and postcontrast acquisitions did not change (the mice were placed exactly at the same position and were not removed from the animal holder).

### Competition experiments

When appropriate, mice received an intravenous injection of anti–VCAM-1 antibody [2.5 mg/kg, clone A(429)] or control isotype IgG 15 min before M3P@αVCAM-1 to saturate the binding sites of the contrast agent.

### Histology

Mice were perfused transcardially with heparinized saline and 4% phosphate-buffered PFA, and the different organs were harvested. Then, the organs were washed three times in 20% sucrose in veronal buffer and embedded in a Cryomatrix (Tissue-Tek OCT) before cryostat sectioning. For iron oxide detection, tissue sections (thickness, 10 μm) were subjected to Prussian blue iron kit (Perl’s staining, Leica Biosystems). Briefly, tissue sections were rehydrated in distilled water for 15 min and covered with a mix of potassium hexacyanoferrate and hydrochloric acid (50/50%) for 20 min. The sections were washed in distilled water and counterstained using nuclear fast red. They were then dehydrated in a gradient bath of ethanol (70, 95, and 100%) and mounted with synthetic resin (EUKITT, Dutscher). For morphology evaluation, we used a ready-to-use kit for hematoxylin and eosin staining (BioGnost). Briefly, frozen sections were successively rehydrated in deionized water, stained using hematoxylin reagent, rinsed in deionized water, and counterstained with a nuclear bluing reagent. Then, the sections were dehydrated in a gradient bath of ethanol (70, 95, and 100%) and mounted with synthetic resin. Images were digitally captured using a VS120 Virtual Slide Microscope (Olympus) and visualized with QuPath (v0.2.3).

### Immunofluorescence

Mice were intracardially perfused with heparinized saline and 4% phosphate-buffered PFA, and the brain and liver were harvested. The organs were cryoprotected in 20% PBS sucrose for 24 hours and embedded in a Cryomatrix (Tissue-Tek OCT) before cryostat sectioning. Cryostat-cut sections (thickness, 10 μm) were collected on polylysine slides and stored at –80°C before processing. Sections were coincubated overnight with rat anti-mouse CD68 (1:800; Abcam, 53444), goat anti-mouse collagen IV (1:1000; SouthernBiotech, 1340), and rat anti-mouse VCAM-1 (1:500; BD Bioscience, 550547). Primary antibodies were revealed by using Fab′2 fragments of donkey anti-rabbit linked to Cy3, anti-rat linked to Cy3, and anti-goat IgG linked to FITC (1:600; Jackson ImmunoResearch, West Grove, USA). Washed sections were coverslipped with antifade medium containing DAPI. Epifluorescence images (~10 images per section) were digitally captured using a Leica DM6000 epifluorescence microscope coupled CoolSNAP camera, visualized with Leica MM AF 2.2.0 software (Molecular Devices, USA), and further processed using ImageJ 1.51k software.

### Statistical analysis

Results are presented as means ± SD. Statistical analyses were performed using Mann-Whitney *U* test. When more than two groups were compared, statistical analyses were performed using Kruskal-Wallis (for multiple comparisons) followed by Mann-Whitney post hoc *U* test. When comparing two groups, a *P* < 0.05 was considered significant (two-sided).
