# Supplementary material for: Tracking the immune response by MRI using biodegradable and ultrasensitive microprobes
Source: Sci Adv. 2022 Jul 13;8(28):eabm3596. doi: 10.1126/sciadv.abm3596 (PMC9278862; doi:10.1126/sciadv.abm3596)
Supplement: Supplementary file 1 — Figs. S1 to S35 [file sciadv.abm3596_sm.pdf]

Science Advances
AAAS

Supplementary Materials for

Tracking the immune response by MRI using biodegradable and ultrasensitive microprobes

Sara Martinez de Lizarrondo *et al.*

Corresponding author: Maxime Gauberti, gauberti@cyceron.fr

Sci. Adv. **8**, eabm3596 (2022)
DOI: 10.1126/sciadv.abm3596

The PDF file includes:

Figs. S1 to S35
Legends for movies S1 to S5

Other Supplementary Material for this manuscript includes the following:

Movies S1 to S5

SUPPLEMENTARY FIGURES

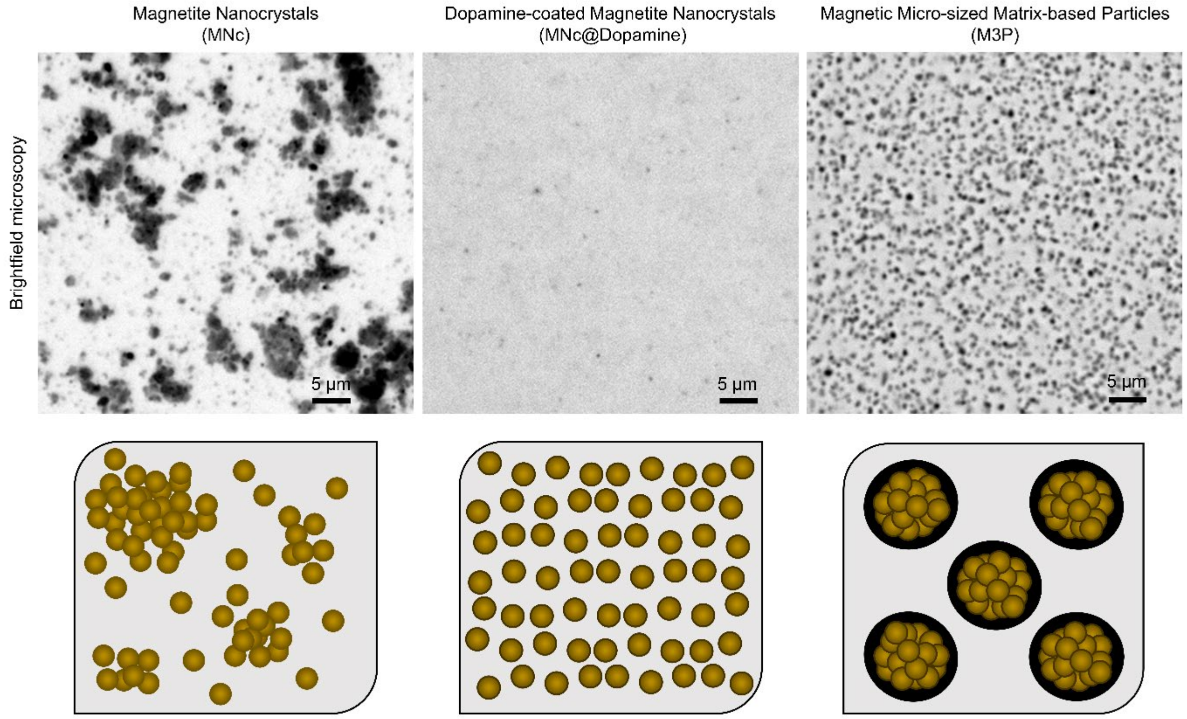

Figure S1. Different steps of the synthesis of M3P. Top: representative brightfield microscopy images of Magnetite Nanocrystals (MNc, left), Dopamine-coated magnetite nanocrystals (MNc@Dopamine, middle) and Magnetic Micro-sized Matrix-based Particles (M3P, right). Bottom: schematic representation of the corresponding images.

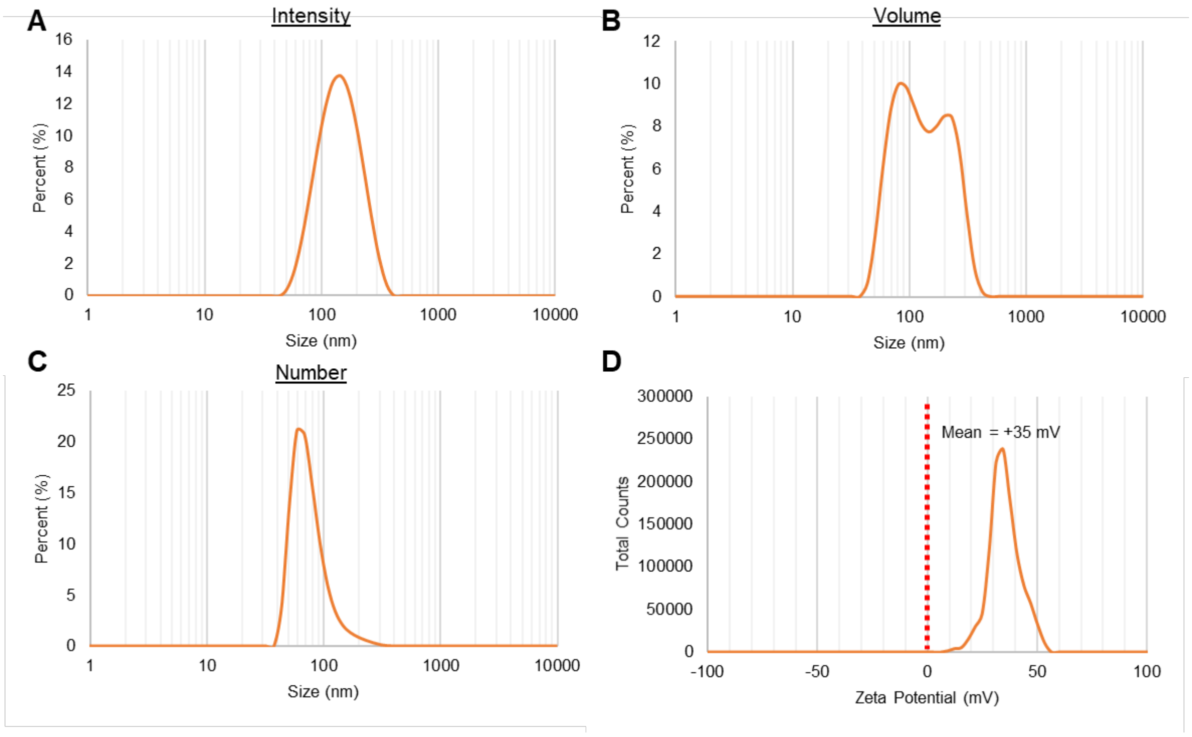

Figure S2. Dynamic light scattering and zeta potential measurements of a representative batch of MNc@Dopamine. (A) Intensity-weighted histogram. (B) Volume-weighted histogram. (C) Number-weighted histogram. (D) Zeta-potential histogram (measured in NaCl 1 mM at pH 7.4). Mean hydrodynamic diameter was 128 nm and polydispersity index was 0.14 for this batch.

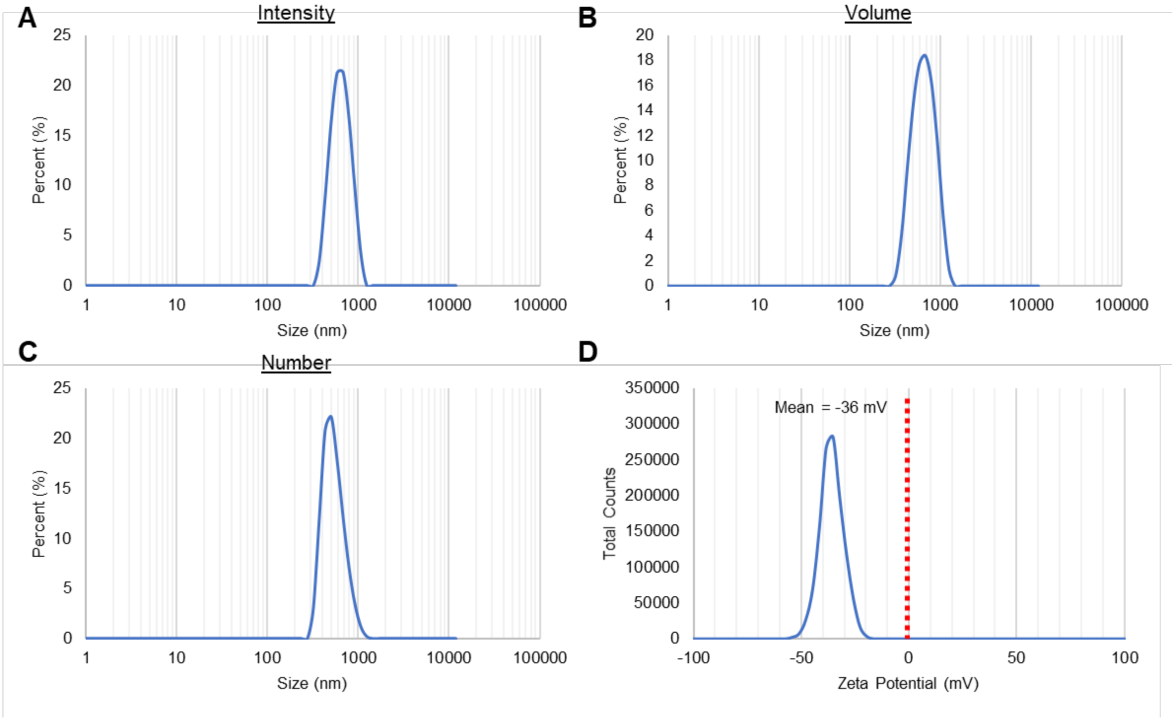

Figure S3. Dynamic light scattering and zeta potential measurements of a representative batch of large 700 nm M3P. (A) Intensity-weighted histogram. (B) Volume-weighted histogram. (C) Number-weighted histogram. (D) Zeta-potential histogram. Mean hydrodynamic diameter was 661 nm and polydispersity index was 0.02 for this batch.

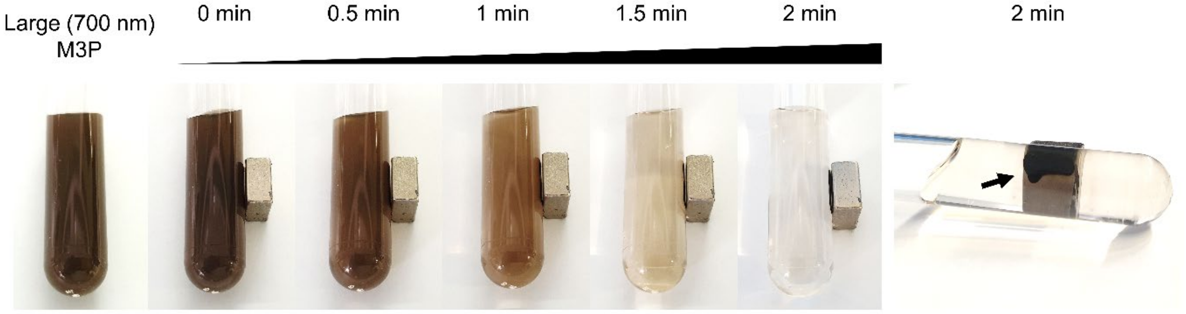

Figure S4. Large M3P are rapidly separable from an aqueous solution by a bench magnet. Pictures of a M3P solution before and at different time points after application of a magnet on the side of the tube.

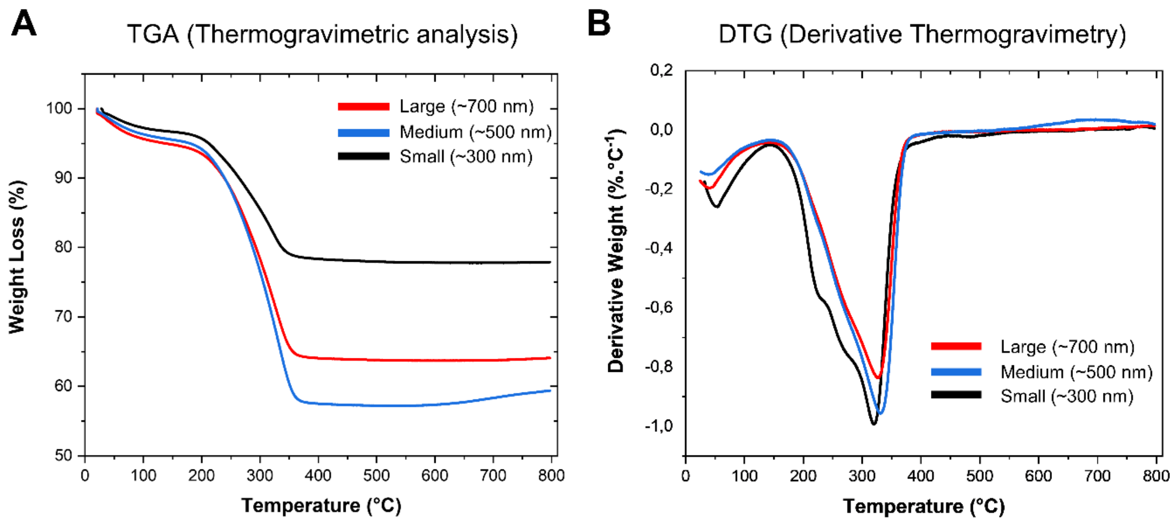

Figure S5. Thermogravimetric analysis of M3Pat different sizes (small, medium, large). (A) Thermogravimetric analysis of M3P at different sizes. (B) Derivative thermogravimetry of M3P at different sizes.

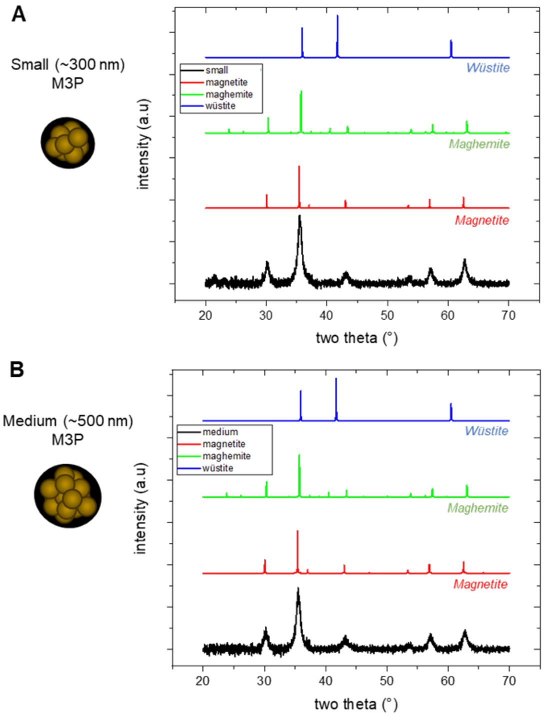

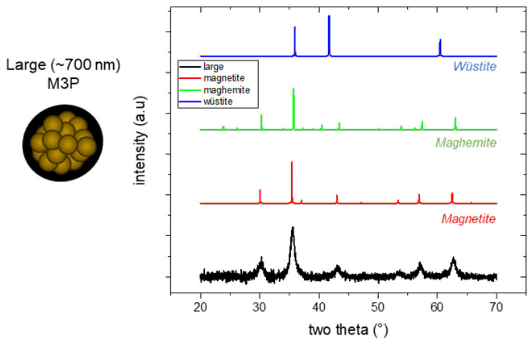

D

Samples	Small (~300 nm)	Medium (~500 nm)	Large (~700 nm)
Lattice parameter (Å)	8.376	8.388	8.376
Crystallite size (nm)	11.0	11.0	11.2

Figure S6. X-ray diffraction (XRD) patterns of M3P at different sizes. XRD patterns of small (A), medium (B) and large (C) M3P. (D) Lattice parameters and crystallite sizes derived from the XRD data.

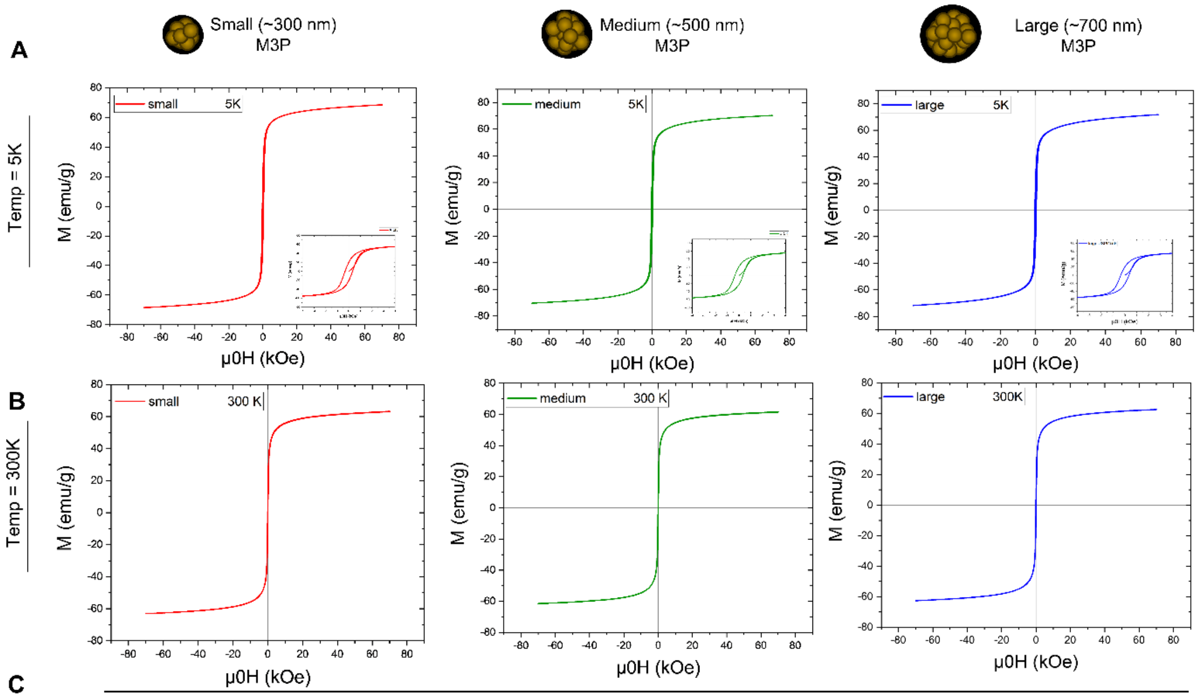

C

	Small (~300 nm)	Medium (~500 nm)	Large (~700 nm)
Hc (Oe) at 5K	323.5 +/- 4.5	344.5 +/- 3.5	344.5 +/- 3.5
Hc (Oe) at 300K	-	-	-
Ms (emu/g) at 5K	67.8 +/- 0.2	69.5 +/- 0.2	68.3 +/- 0.3
Ms (emu/g) at 300K	69.0 +/- 0.2	68.8 +/- 0.4	65.3 +/- 3.7

Figure S7. Superconducting quantum interference device (SQUID) magnetometry of M3P at different sizes. (A) Hysteresis cycles at 5K of small (left), medium (middle) and large (right) M3P. (B) Hysteresis cycles at 300K of small (left), medium (middle) and large (right) M3P. (C) Summary of the results. Hc: coercive field. Ms: saturation magnetization.

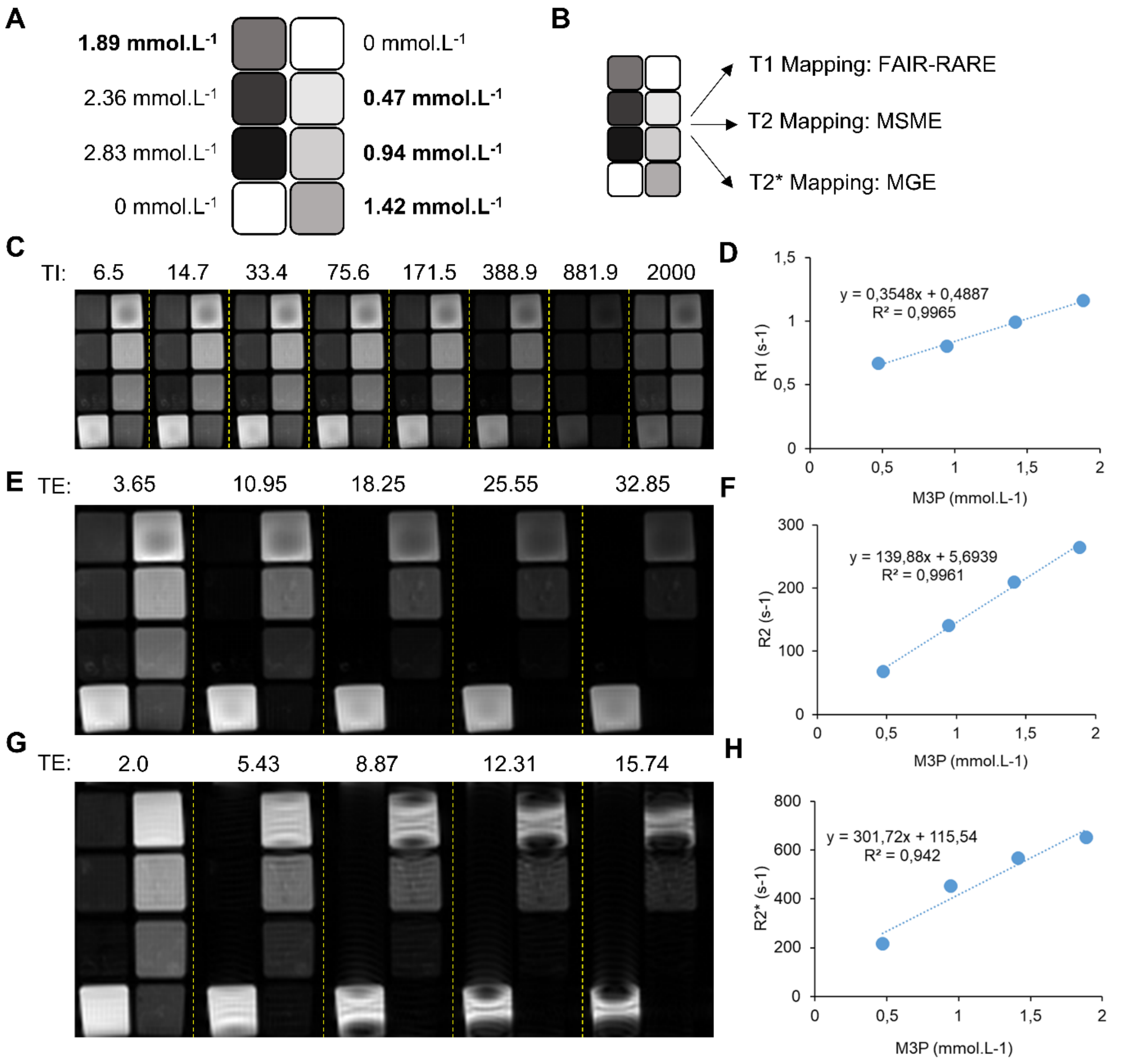

Figure S8. Relaxivity measurement of large 700 nm M3P using a 7T preclinical MRI. (A) Schematic representation of the multi-well plate containing M3P embedded in 2% agarose gels at different concentrations. In bold are the wells that were used for computing relaxivity values. This concentration range was selected to provide a linear relationship between relaxivity values and M3P concentrations. (B) Schematic representation of the experiments. (C) Representative FAIR-RARE images at different inversion time (TI). (D) Corresponding computation of the R1 relaxivity of the particles. (E) Representative multi-echo (MSME) images at different echo times (TE). (F) Corresponding computation of the R2 relaxivity of the particles. (G) Representative multi-gradient echo (MGE) images at different TE. (H) Corresponding computation of the R2* relaxivity of the particles.

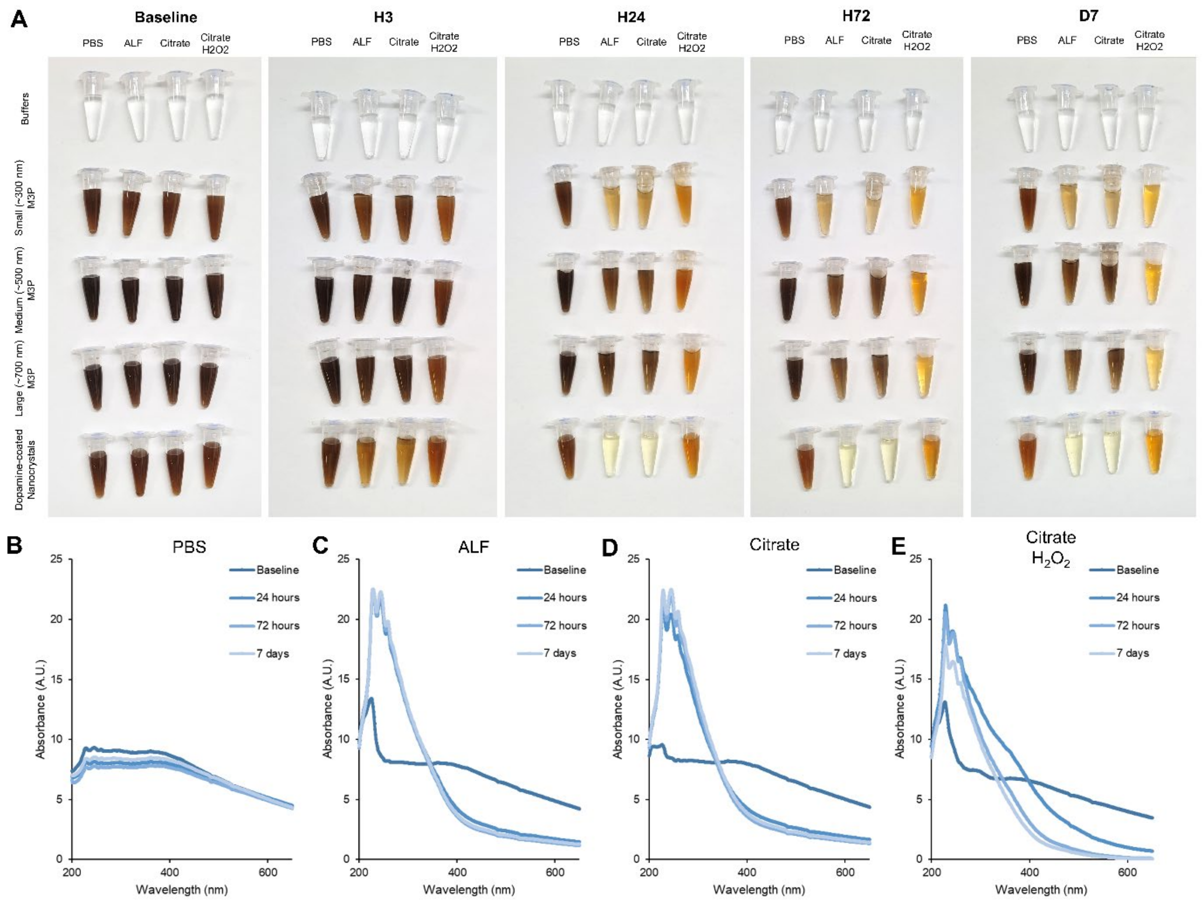

Figure S9. Degradation of large M3P in different buffers as assessed by visual inspection and UV-Visible spectroscopy. (A) Pictures of different solutions containing either M3P at different sizesor Dopamine-coated Nanocrystals (0.5 mg/ml equivalent of iron) in phosphate buffered saline (PBS), artificial lysosomal fluid (ALF), citrate and citrate with hydrogen peroxide (H_2O_2) at different time points after incubation at 37°C under mild agitation. (B-E) UV-Vis spectroscopy of the large M3P (700 nm) in PBS (B), ALF (C), citrate (D) and citrate with H2O2 (E) at different time points.

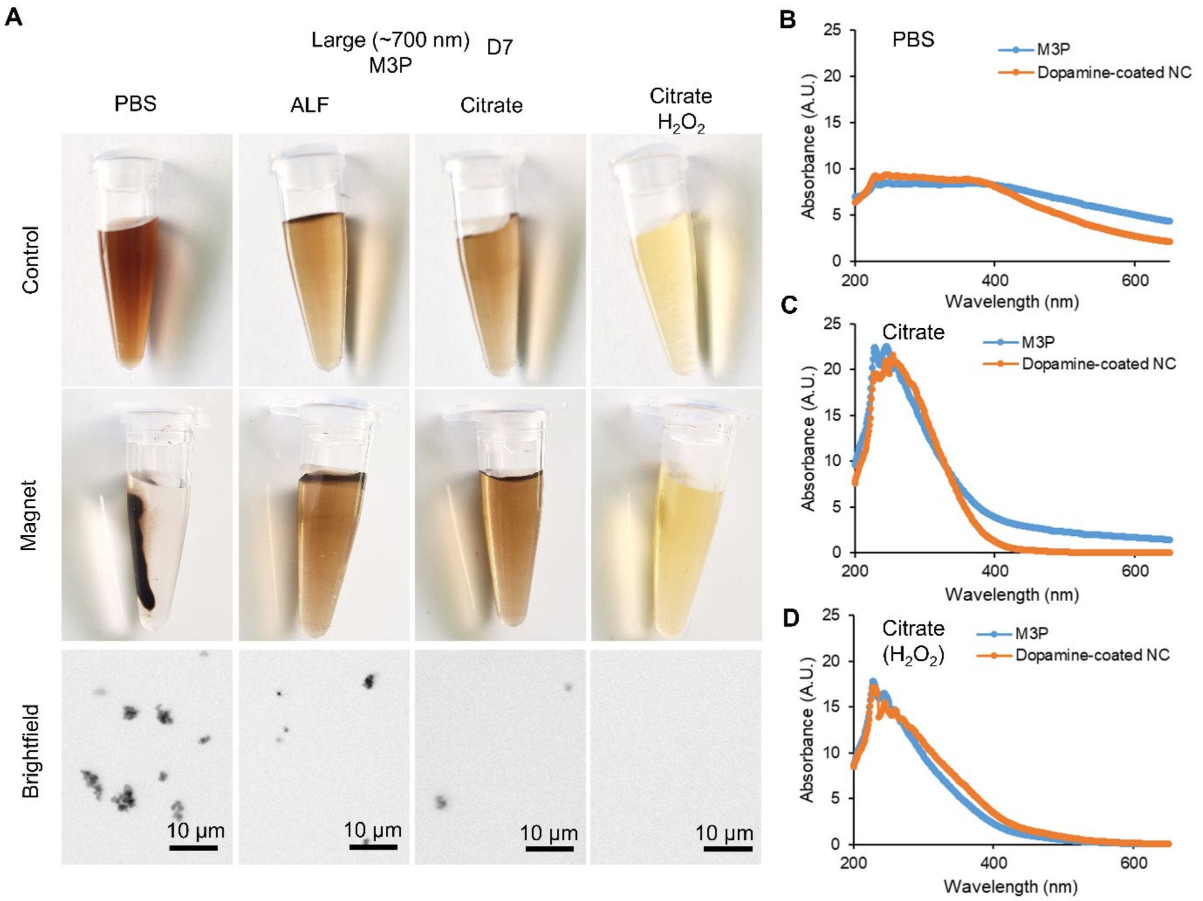

Figure S10. Large M3P are completely degraded after 7 days of incubation in citrate buffer with hydrogen peroxide. (A) Pictures of solutions containing large M3P in phosphate buffered saline (PBS), artificial lysosomal fluid (ALF), citrate and citrate with hydrogen peroxide (H_2O_2). (B-D) UV-Vis spectroscopy of the large M3P (700 nm) and Dopamine-coated Nanocrystals (NC) in PBS (B), citrate (C) and citrate with H2O2 (D).

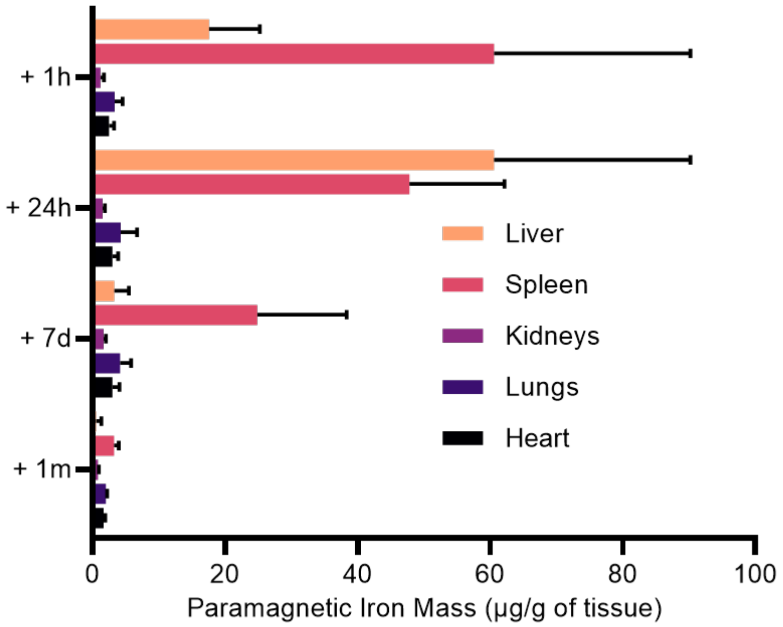

Figure S11. Mass of paramagnetic iron per gram of tissue in the liver, spleen, kidneys, lungs, and heart at different time points after intravenous injection of 4 mg/kg large M3P, as assessed by electronic paramagnetic resonance. (n= 5 per group)

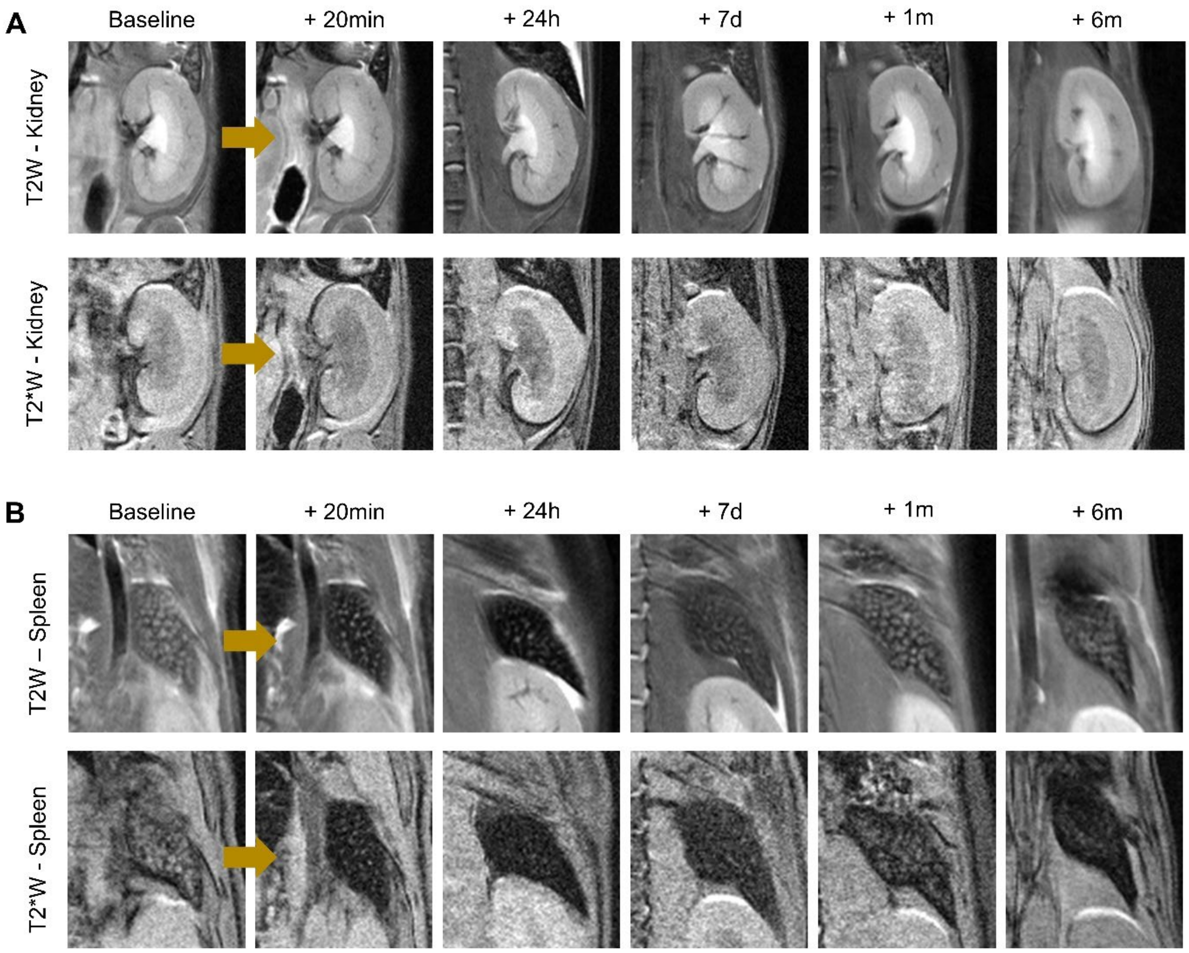

Figure S12. Longitudinal MRI of the kidneys and spleen after intravenous injection of M3P. (A) Representative longitudinal T2-weighted images of the kidneys at baseline and at different time points after intravenous injection of 4 mg/kg large M3P. (B) Representative longitudinal T2-weighted images of the spleen at baseline and at different time points after intravenous injection of 4 mg/kg large M3P.

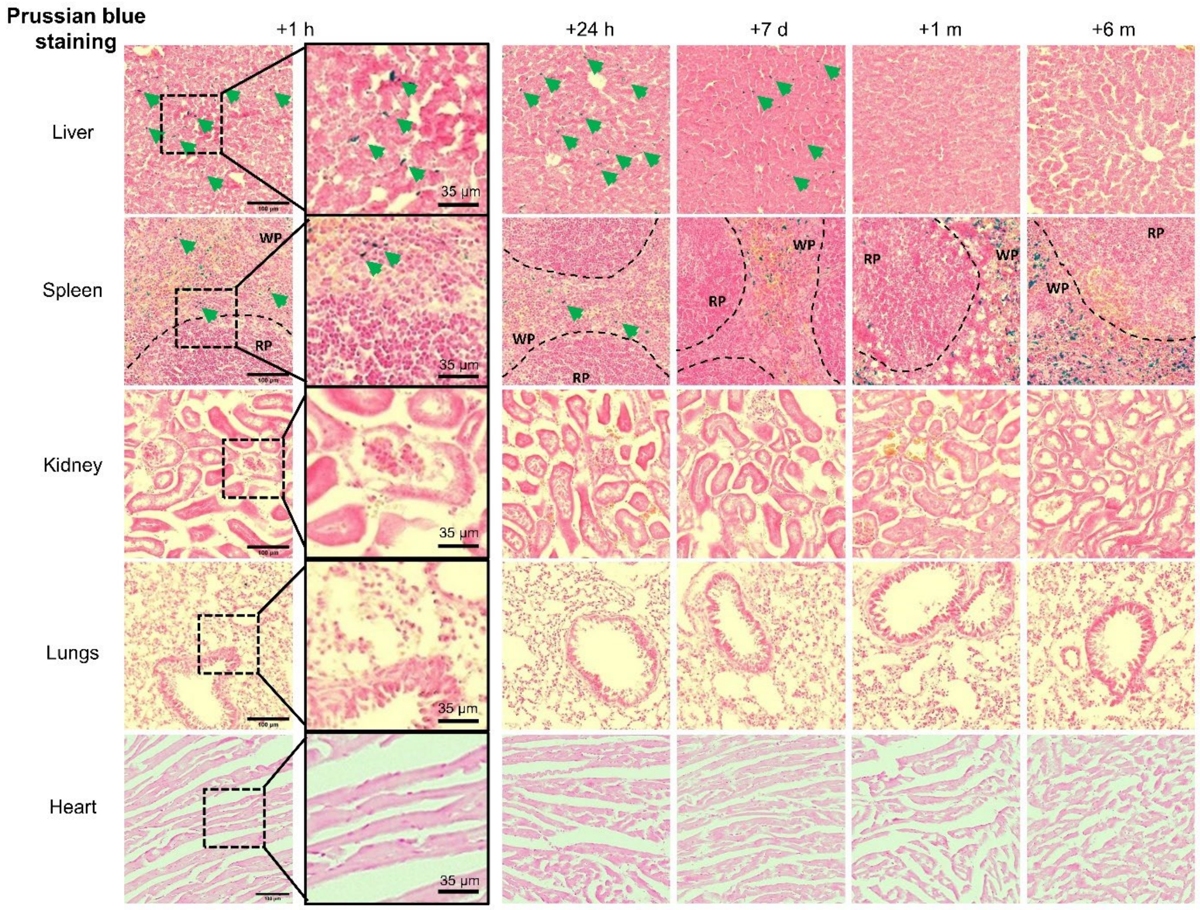

Figure S13. Longitudinal follow up of M3P degradation by Prussian blue staining. Representative histological images of the liver, spleen, kidney, lungs and heart with Prussian blue staining at different time point after intravenous injection of 4 mg/kg of M3P. M3P were detected at early time points in the liver (arrowheads). The presence of iron in the white pulp of the spleen is physiologic and corresponds to siderophages with intracytoplasmic iron. No particle was detected after 1 month in any organs. Images are representative of n=6 animals per time point. WP: white pulp. RP: red pulp.

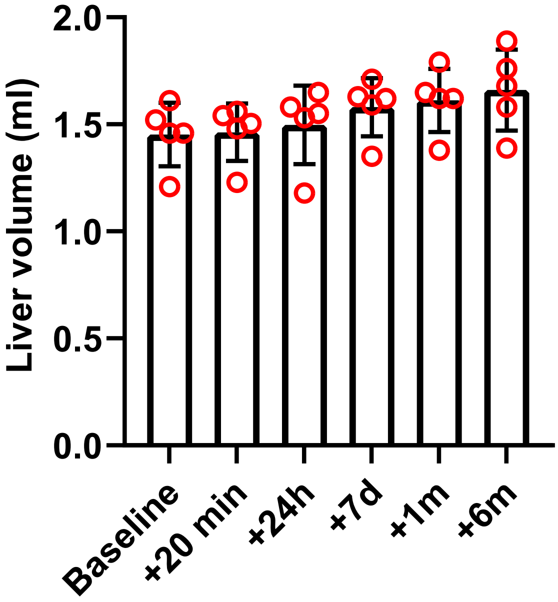

Figure S14. Liver volume of mice at baseline and at different time points after intravenous injection of large M3P (4 mg/kg) as assessed by MRI.

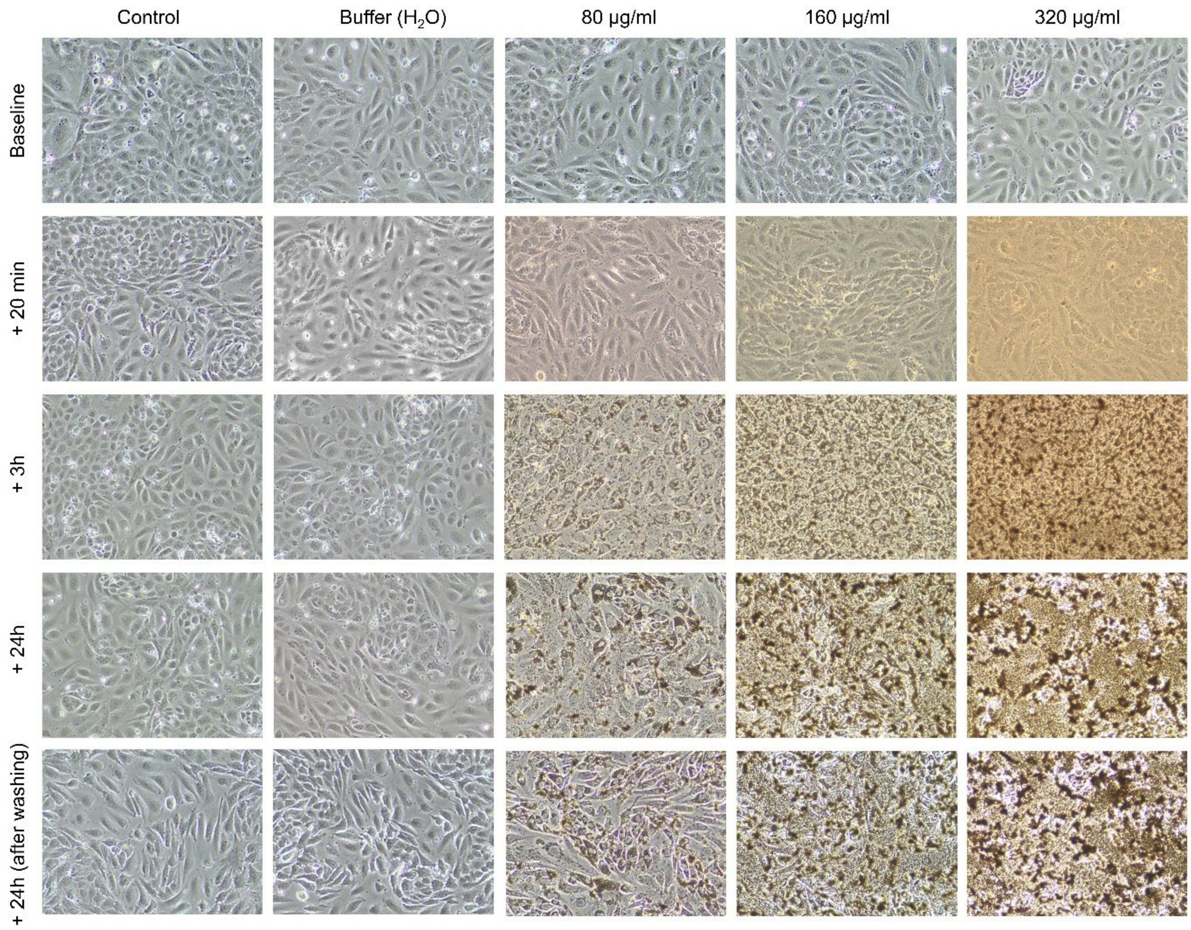

Figure S15. Representative histological images of human venous endothelial cells (HUVEC) before and at different time points after incubation with different concentrations of large M3P.

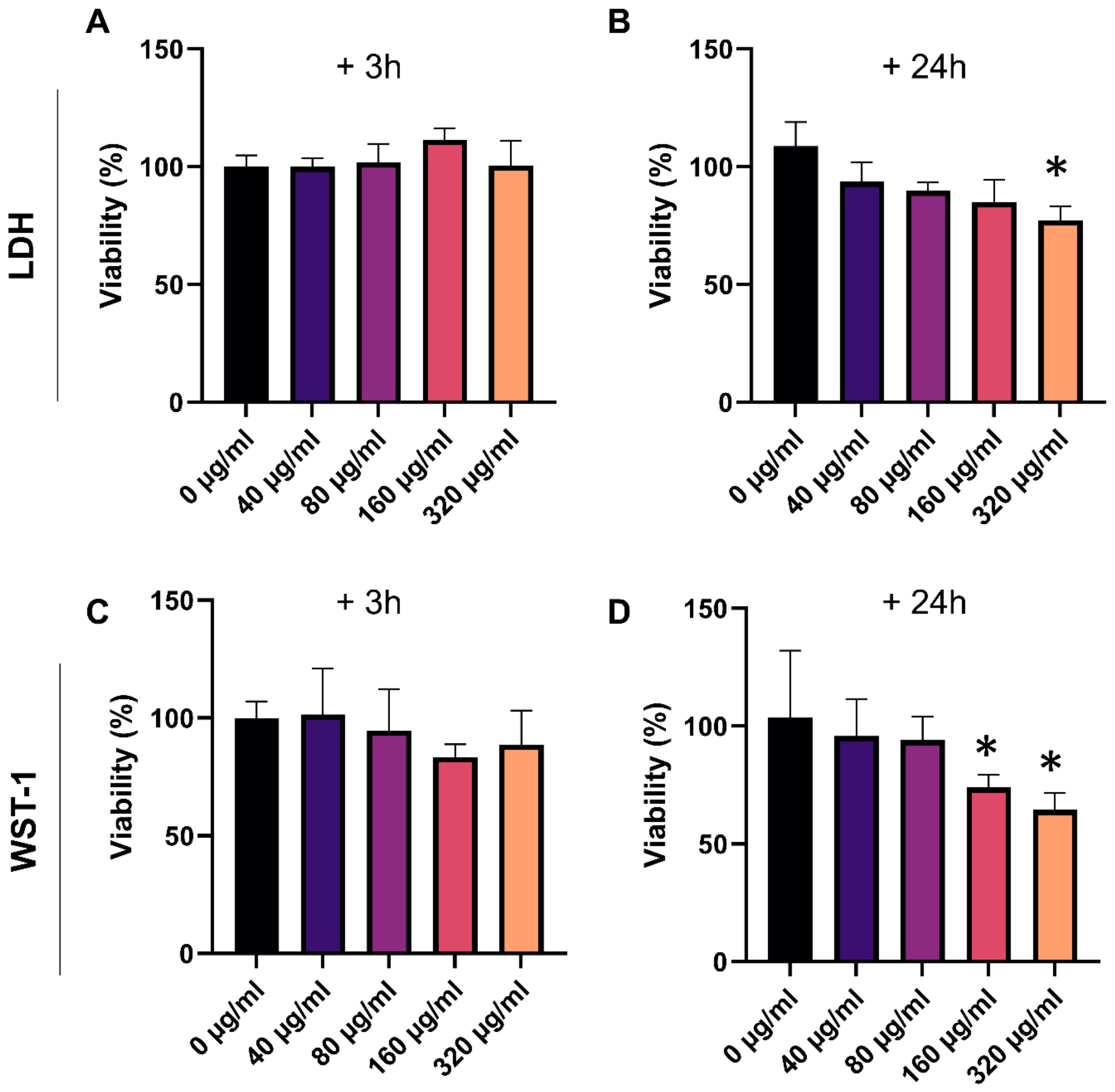

Figure S16. Cytotoxicity of large M3P on human umbilical vein endothelial cells (HUVEC). (A-B) Viability of HUVEC cells 3 hours (A) and 24 hours (B) after incubation with different concentrations of large M3P as assessed by the lactate dehydrogenase (LDH) assay (n=5 per group). (c-d) Viability of HUVEC cells 3 hours (C) and 24 hours (D) after incubation with different concentrations of large M3P as assessed by the Water Soluble Tetrazolium-1 (WST-1) assay (n=5 per group). *$p<0.05$ compared to the control group (0 µg/ml).

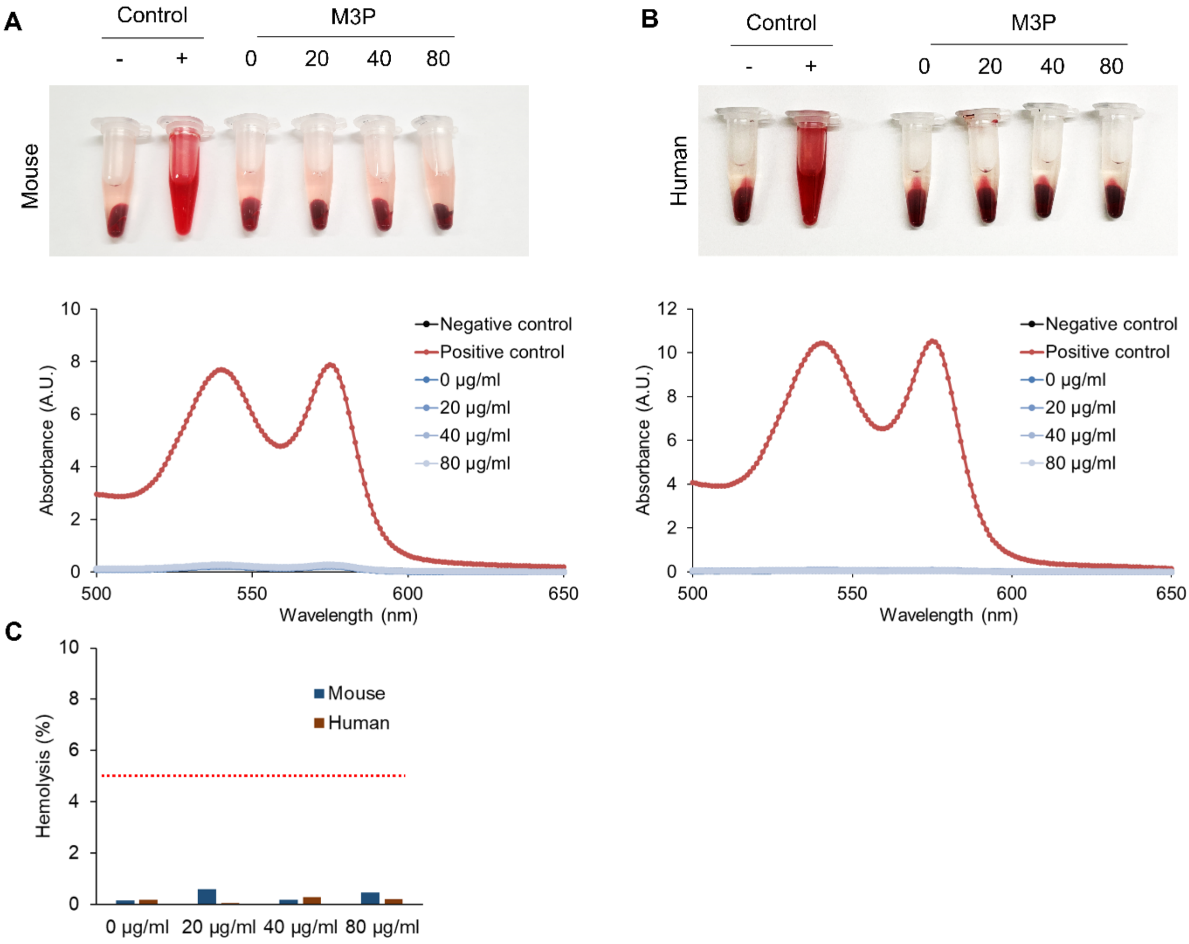

Figure S17. Hemolysis assays in mouse and human blood. (A) Top: pictures of test tubes containing mouse blood after incubation with different concentrations of large M3P and centrifugation. Bottom: Spectroscopy of the supernatant of the different tubes. (B) Top: pictures of test tubes containing human blood after incubation with different concentrations of large M3P and centrifugation. Bottom: Spectroscopy of the supernatant of the different tubes. (C) Summary of the hemolysis rate for each condition measured by absorbance at 540 nm (representative of n=3 per group). No condition exceeded 5% of hemolysis, the critical safe hemolytic ratio for biomaterials according to ISO/TR 7406.

A

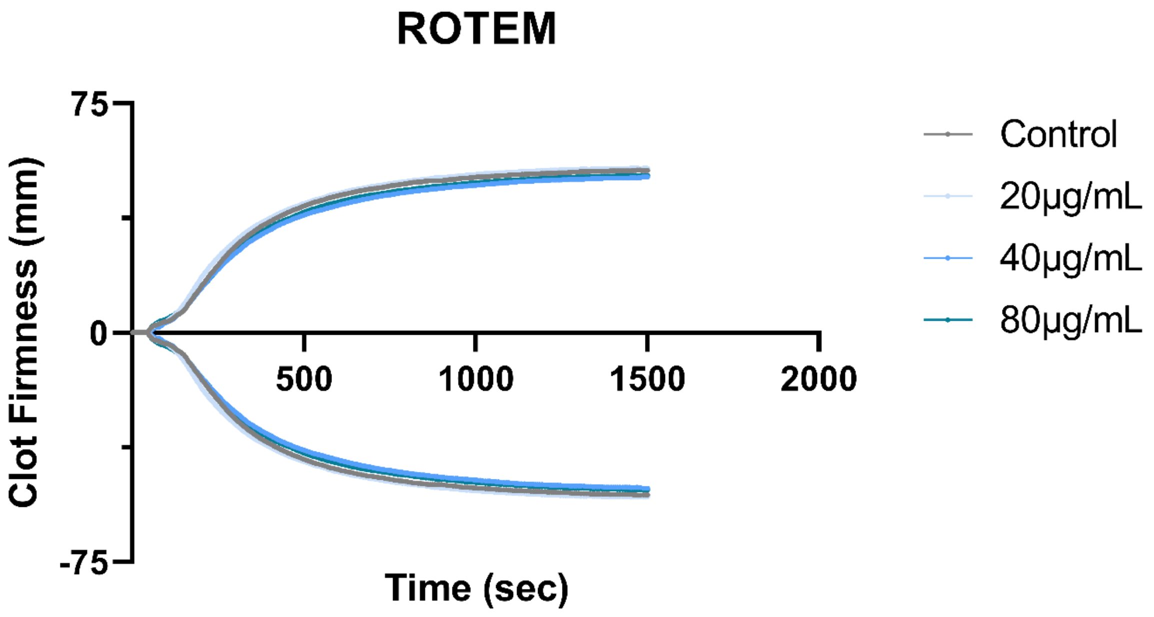

B

	CT (s)		A10		CFT (s)		MCF (mm)		α angle (°)	
	Mean	SD	Mean	SD	Mean	SD	Mean	SD	Mean	SD
**Control**	50.0	6.1	42.0	6.2	204.0	35.3	50.7	5.1	57.7	9.1
**M3P 20ug/mL**	56.3	8.7	43.3	5.7	181.3	32.5	51.3	4.7	57.0	5.3
**M3P 40ug/mL**	54.7	4.7	41.7	4.9	195.0	32.1	49.3	3.8	54.3	4.6
**M3P 80ug/mL**	49.7	2.1	42.0	6.9	199.7	39.1	49.7	5.8	59.0	1.0

Figure S18. Rotational thromboelastometry (ROTEM) in the presence of large M3P in whole human blood. (A) Representative ROTEM curves using the EXTEM assay with different concentrations of large M3P. (B) Corresponding main ROTEM parameters, showing no significant effect of large M3P on clot formation. Clotting time = CT (seconds), Amplitude at 10 minutes = A10, clot formation time = CFT (seconds), maximal clot firmness = MCF (mm), alpha angle = α angle (°).

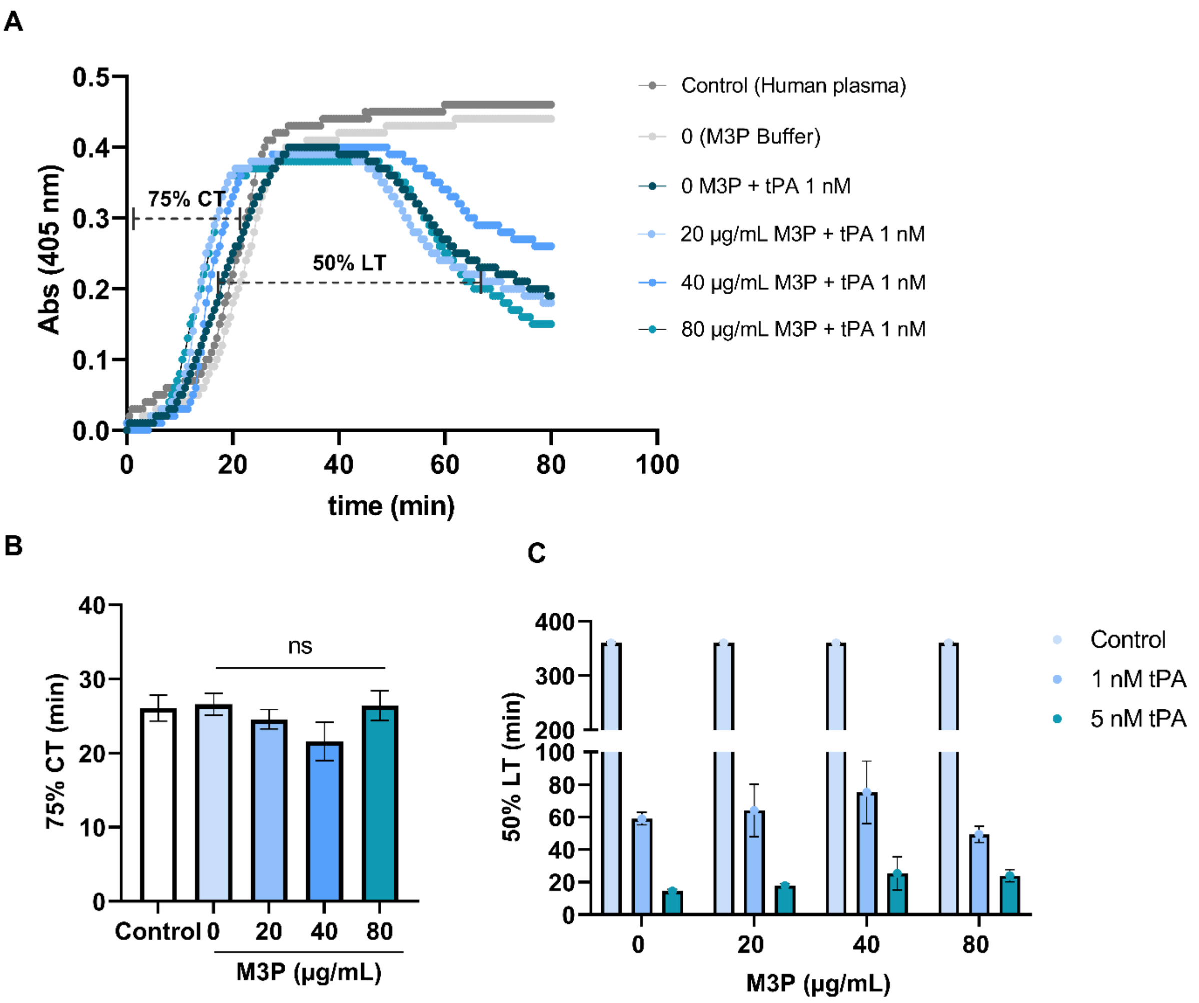

Figure S19. Clot lysis assay using human plasma in the presence of large M3P. (A) Representative clot lysis assay curves showing how 75% clotting time (75%CT) and 50% lysis time (50%LT) were measured. (B) Mean 75%CT of human plasma in the presence of different concentrations of large M3P (n=5 per group). (C) Mean 50%LT of human plasma in the presence of different concentrations of large M3P (n=5 per group). The 50%LT was set to 350 minutes in samples without lysis. ns: not significant.

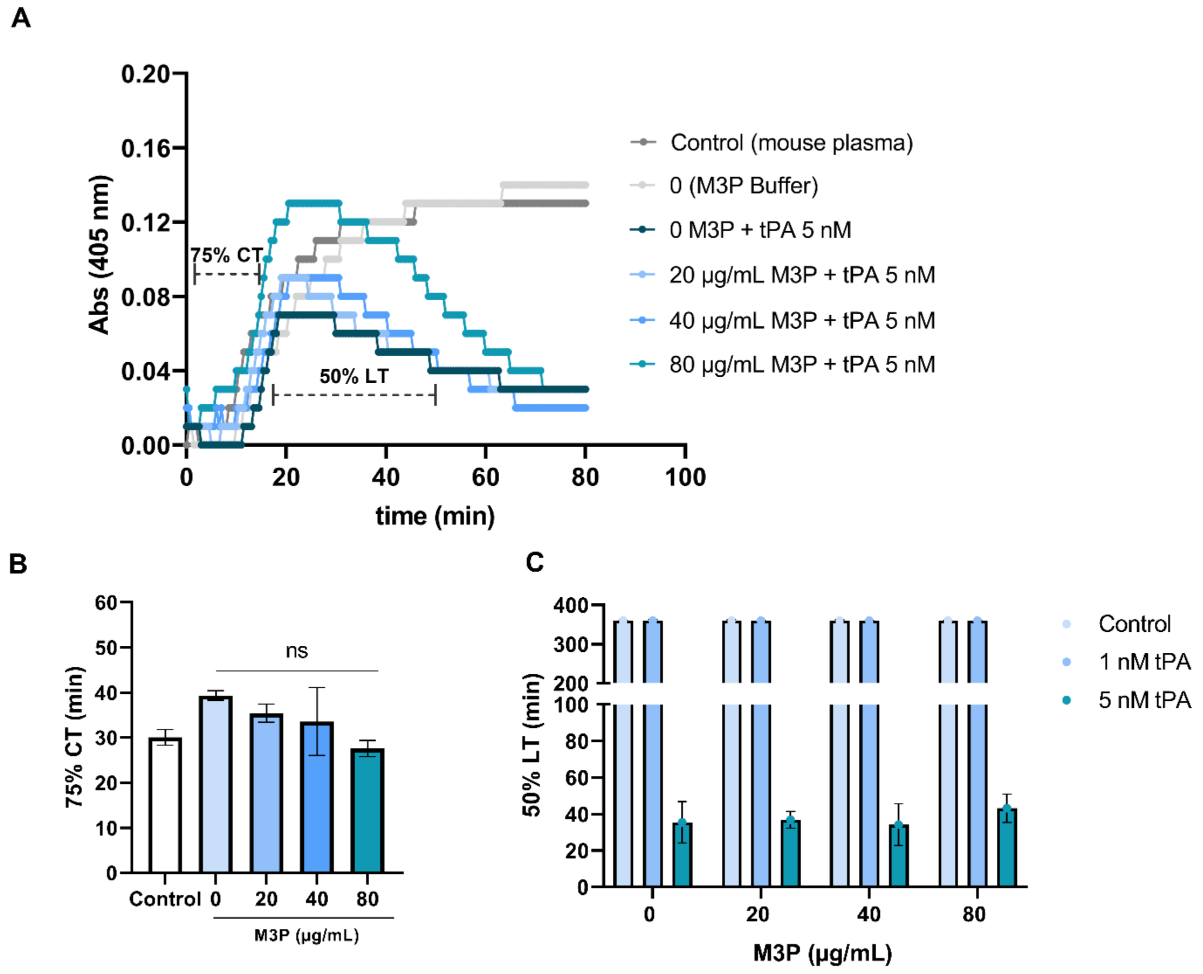

Figure S20. Clot lysis assay using mouse plasma in the presence of large M3P. (A) Representative clot lysis assay curves showing how 75% clotting time (75%CT) and 50% lysis time (50%LT) were measured. (B) Mean 75%CT of mouse plasma in the presence of different concentrations of large M3P (n=5 per group). (C) Mean 50%LT of mouse plasma in the presence of different concentrations of large M3P (n=5 per group). The 50%LT was set to 350 minutes in samples without lysis. ns: not significant.

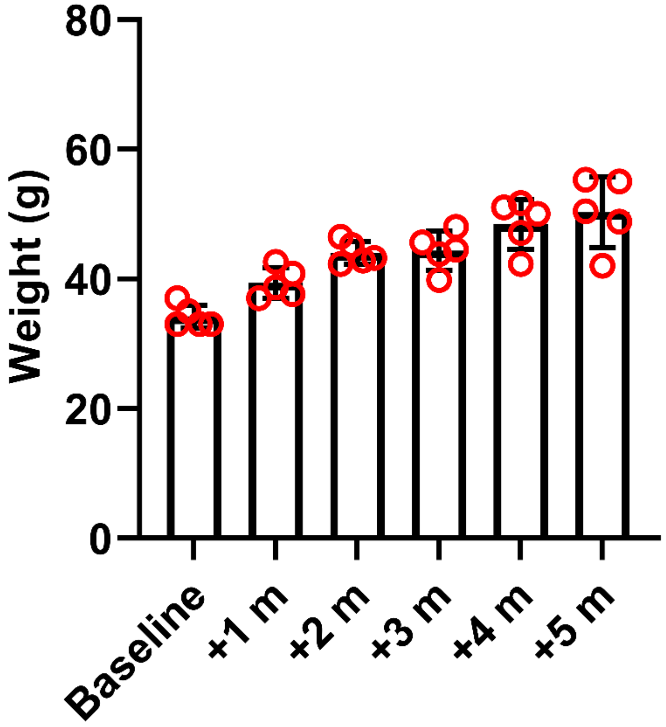

Figure S21. Body weight of mice at baseline and at different time points after intravenous injection of large M3P (4 mg/kg).

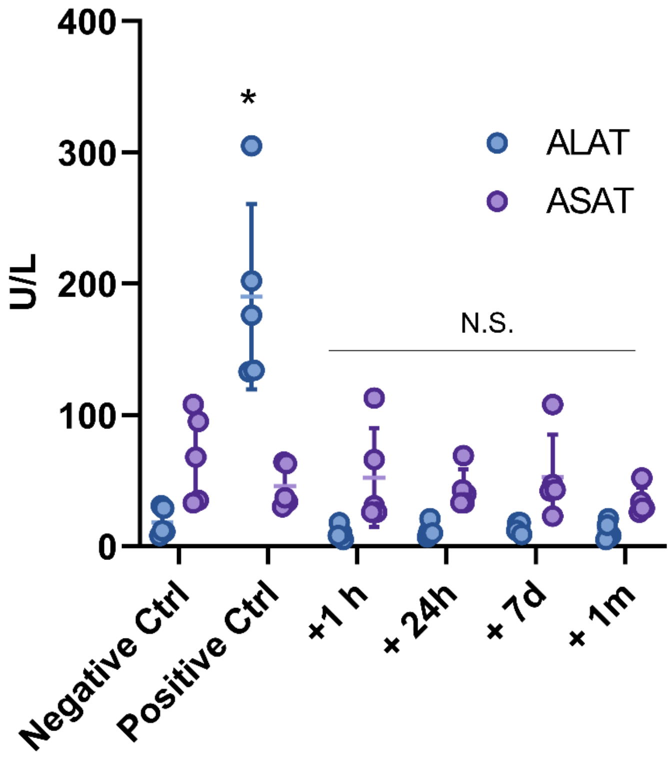

Figure S22. Plasma concentrations of liver enzymes at different time points after intravenous injection of large M3P (4 mg/kg) in mice as assessed by ELISA. The negative control (Negative Ctrl) samples were collected in control mice that did not receive M3P. The positive control (Positive Ctrl) samples were collected 24 hours after intra-peritoneal injection of 5 mg/kg E. coli lipopolysaccharide (LPS). * $p<0.05$ versus negative control. ALAT: Alanine transaminase. ASAT: Aspartate aminotransferase. N.S.: not significant.

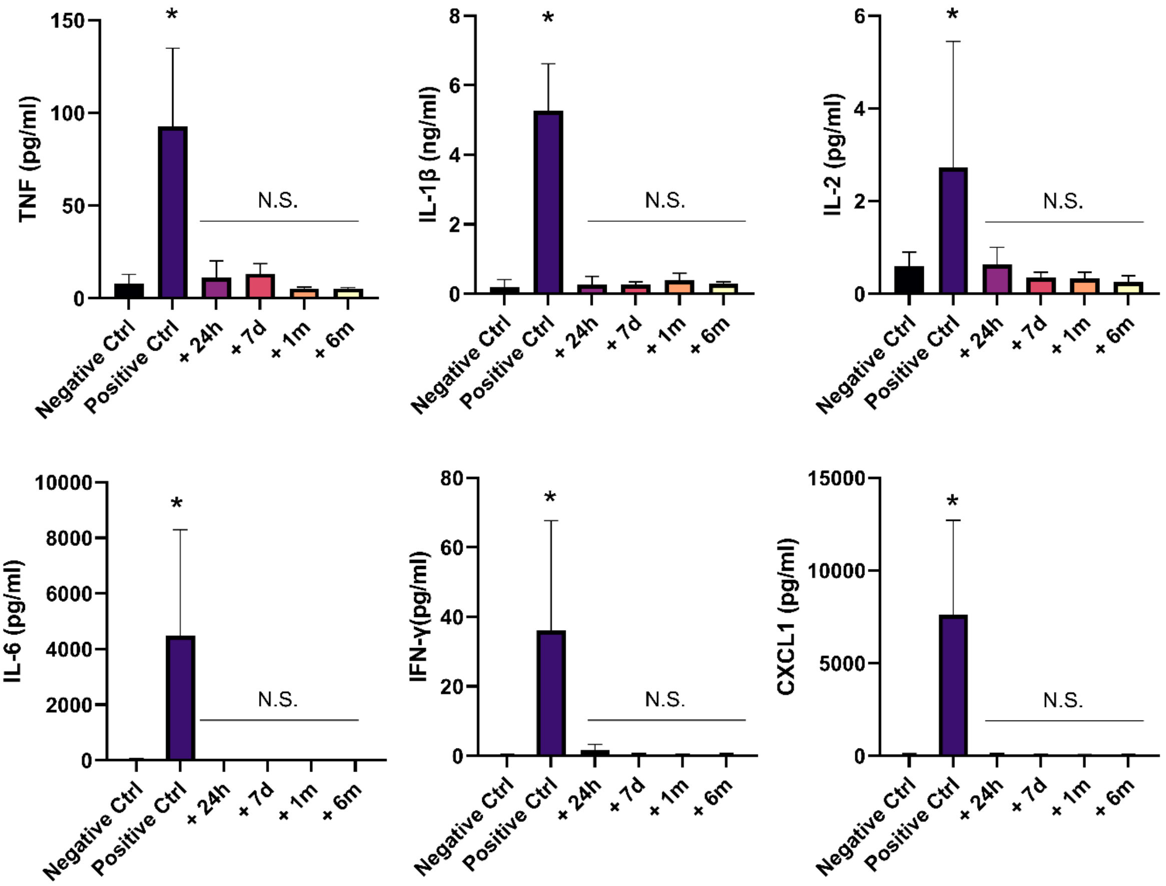

Figure S23. Plasma concentrations of a set of key cytokines and chemokines at different time points after intravenous injection of large M3P (4 mg/kg) in mice as assessed by ELISA. The negative control (Negative Ctrl) samples were collected in control mice that did not receive M3P. The positive control (Positive Ctrl) samples were collected 24 hours after intra-peritoneal injection of 5 mg/kg E. coli lipopolysaccharide (LPS). *$p<0.05$ versus negative control. N.S.: not significant. TNF: tumor necrosis factor. IL: Interleukin. IFN: interferon. CXCL1: chemokine (C-X-C motif) ligand 1.

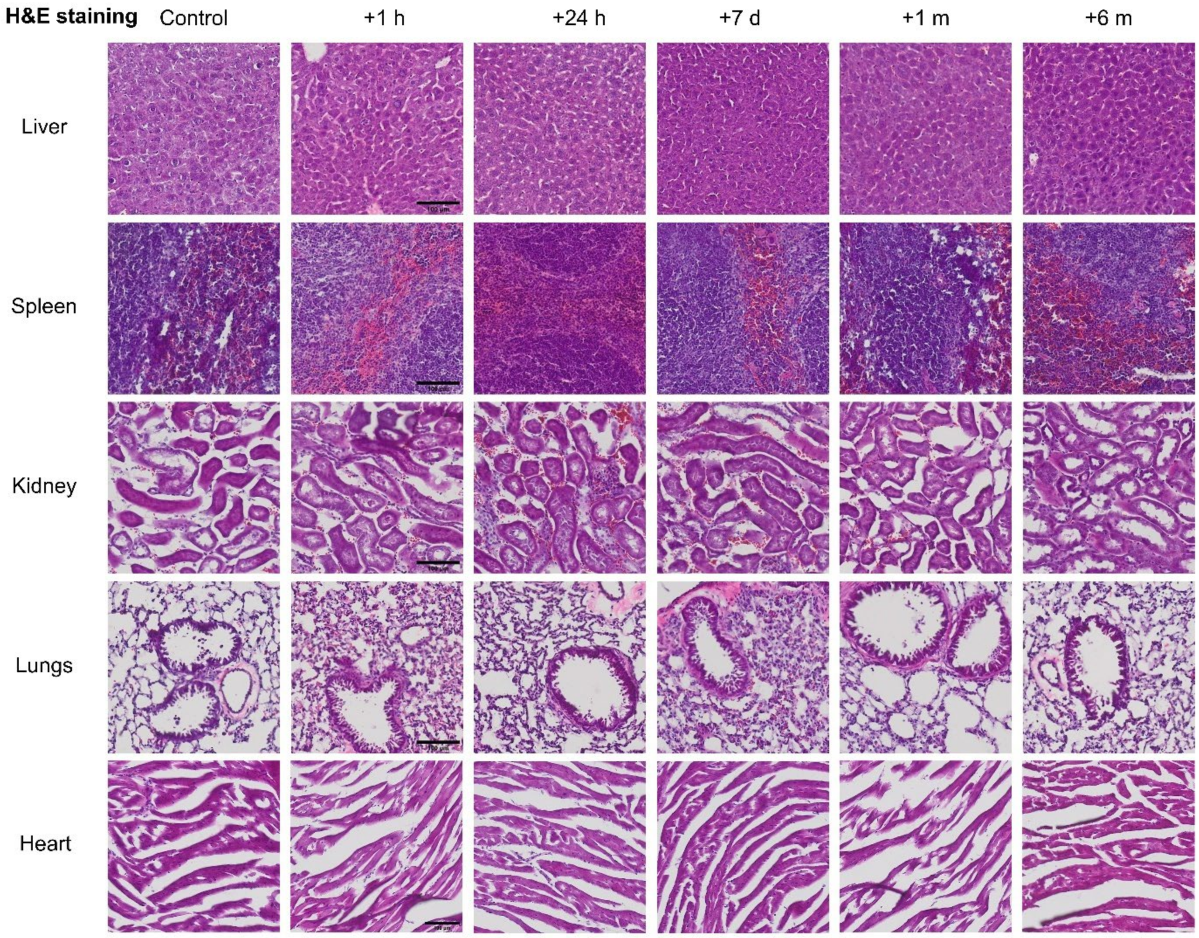

Figure S24. Longitudinal evaluation of tissue histology by hematoxylin eosin staining (H&E) before and after intravenous injection of large M3P (4 mg/kg) in mice. Images are representative of n=6 animals per time point.

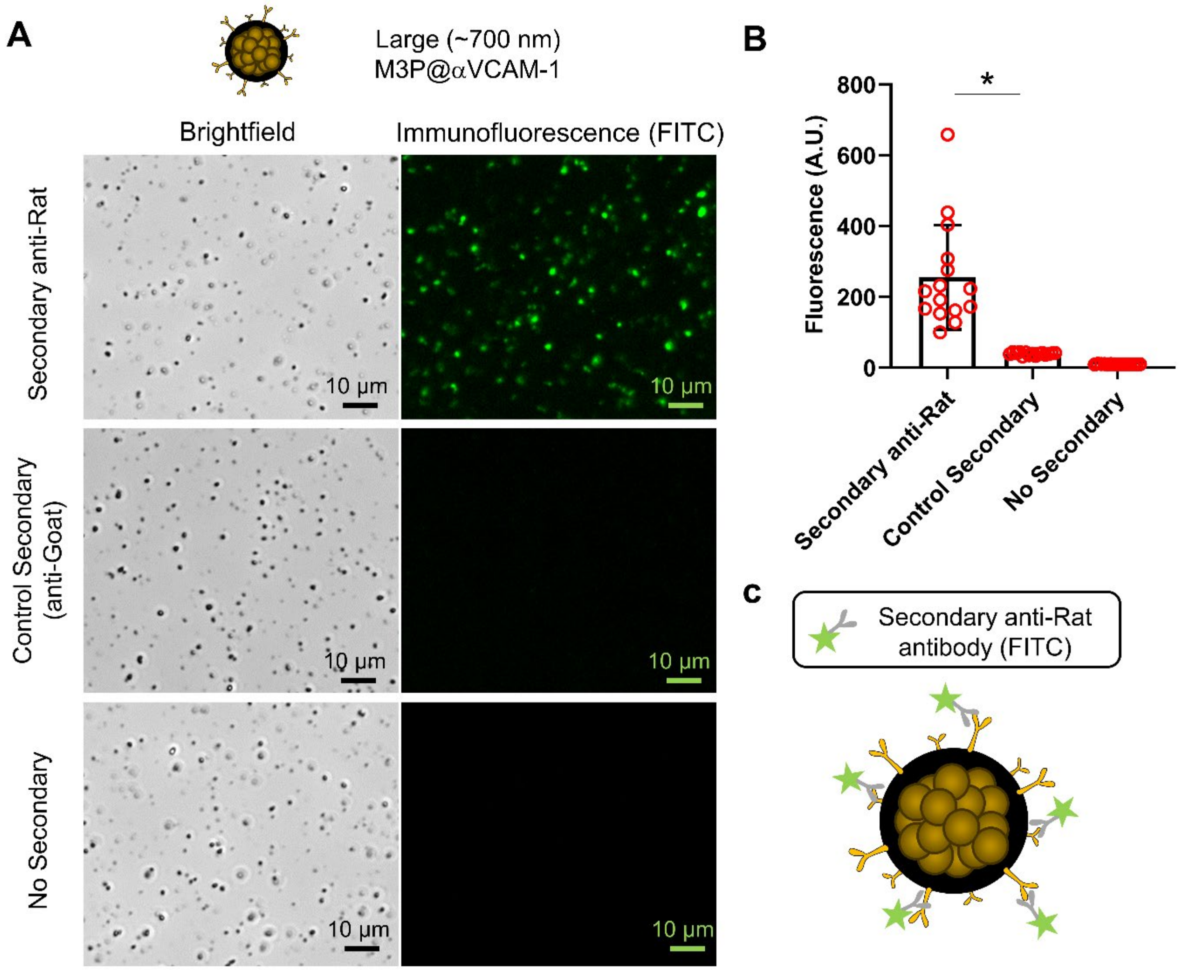

Figure S25. Characterization of large M3P after complexation to monoclonal antibodies targeting mouse vascular cell adhesion molecule-1 (VCAM-1). (A) Top: schematic representation of M3P@αVCAM-1. Bottom: left, representative brightfield microscopic images of the particles; right: representative fluorescence microscopic images of the particles. (B) Corresponding quantification of particle fluorescence according to the type of fluorescent secondary antibodies (n= 25 per group). (C) Schematic representation of the findings, demonstrating the successful conjugation of monoclonal antibodies on the surface of large M3P. * $p<0.05$.

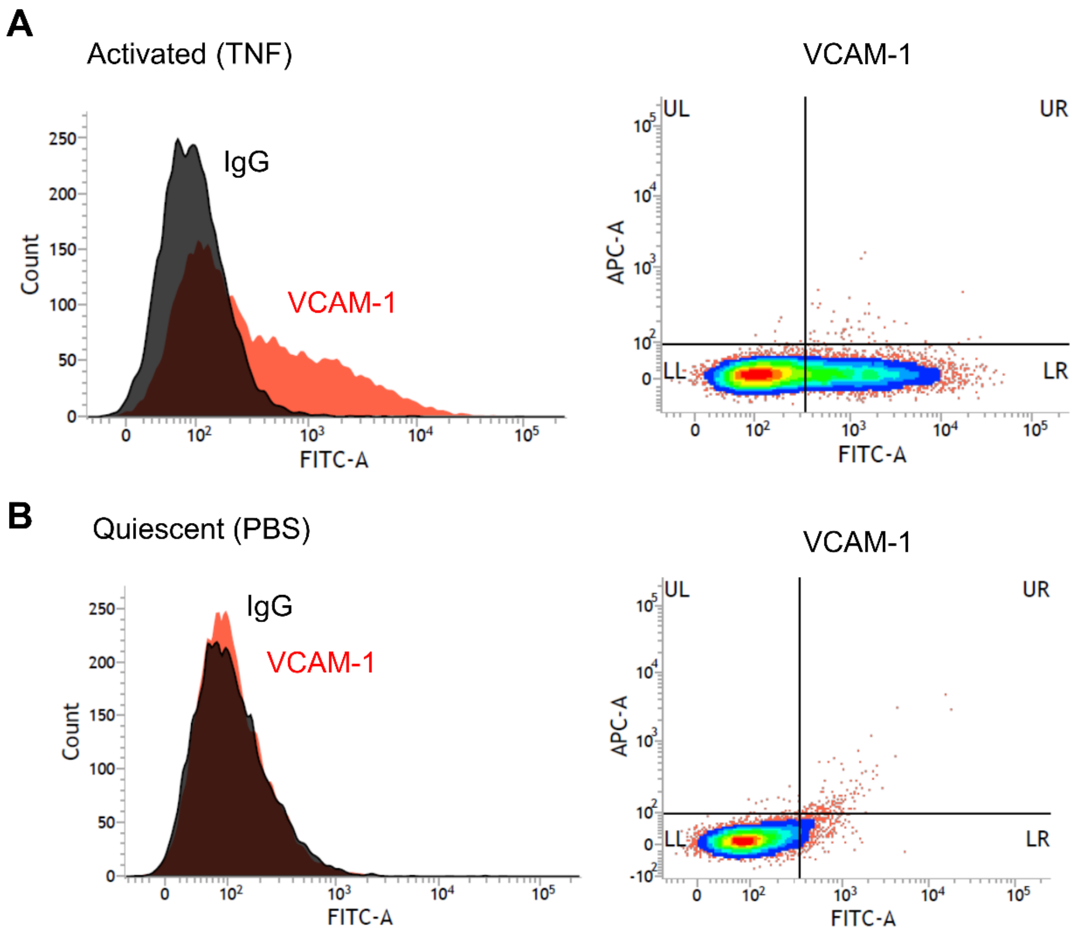

Figure S26. Human brain endothelial cells (HCMECD/3) overexpress VCAM-1 after stimulation with TNF as assessed by flow cytometry. (A) Left: Flow cytometry results using primary anti-VCAM-1 antibody or control immunoglobulin (IgG) after stimulation with TNF (50 ng/ml for 24 hours). Right: corresponding density plot. (B) Same as in (A) using unstimulated cells.

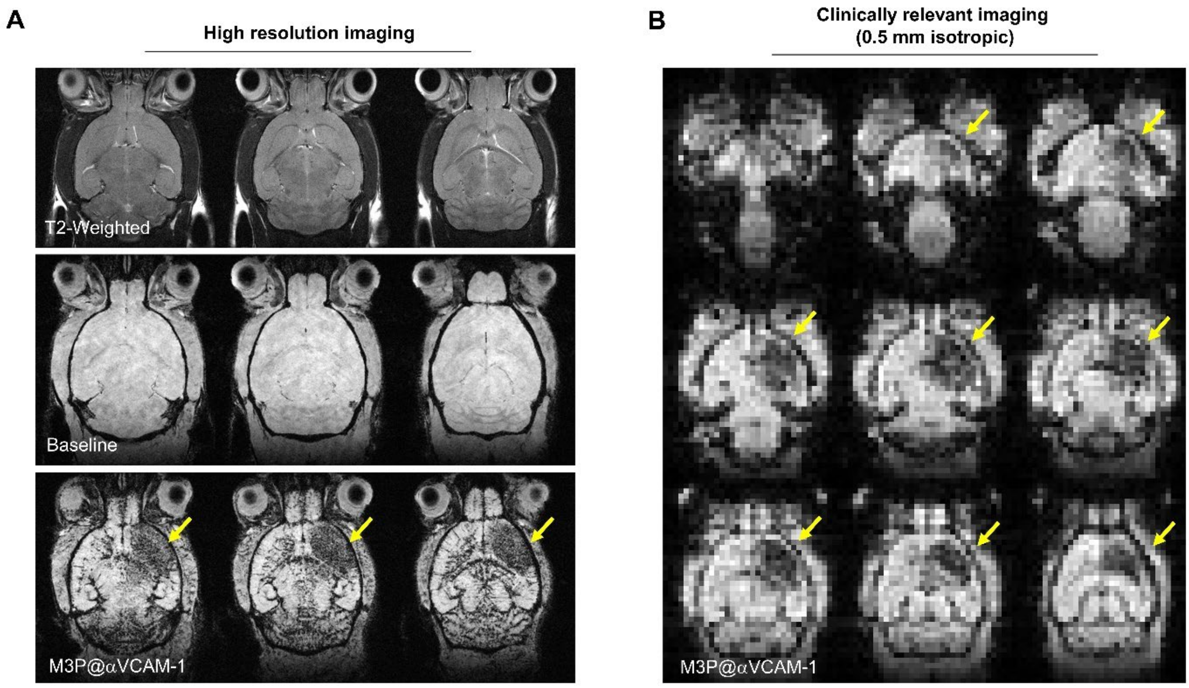

Figure S27. M3P@αVCAM-1 are detectable at clinically relevant spatial resolution. (A) Representative high-resolution MRI of the brain after intrastriatal injection of LPS (1.0 µg). Top: T2-weighted images. Middle: High resolution T2*-weighted images before injection of contrast agent. Bottom: High resolution T2*-weighted images after intravenous injection of M3P@αVCAM-1 (4 mg/kg). (B) Brain MRI at clinically relevant resolution (0.5 mm isotropic) of the same animal as in (A) after intravenous injection of M3P@αVCAM-1 (4 mg/kg). Arrowheads indicate the numerous signal voids in the right striatum after injection of the contrast agent.

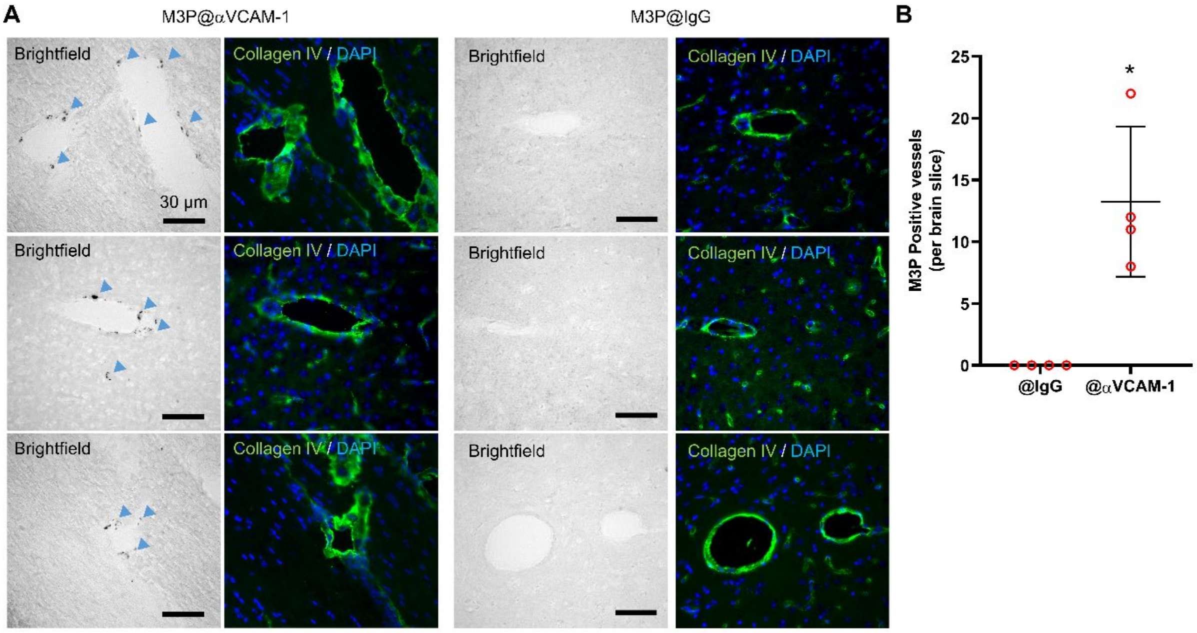

Figure S28. M3P@αVCAM-1 accumulate in the inflamed striatum of LPS treated mice. (A) Representative brightfield and immunofluorescence images of the striatum of mice at 24 hours after intrastriatal LPS injection (1.0 µg) and 60 minutes after intravenous injection of M3P@αVCAM-1. Three images from three different animals are presented for M3P@αVCAM-1 and for control M3P@IgG. (B) Corresponding quantification (n=4 per group). *$p<0.05$.

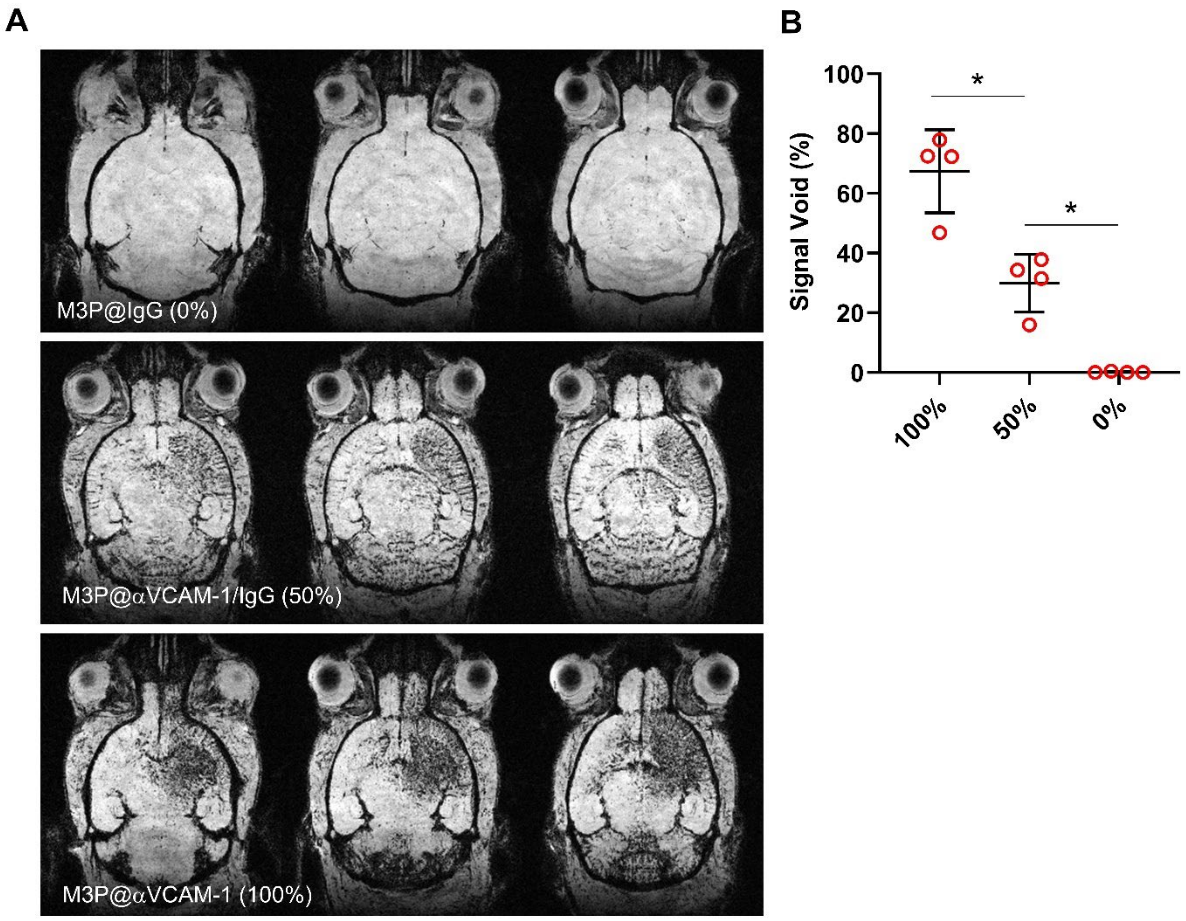

Figure S29. Higher density of anti-VCAM-1 monoclonal antibodies on the surface of M3P improves the sensitivity of molecular MRI. (A) Representative T2*-weighted images of the brain after intrastriatal injection of LPS (1.0 µg) and after intravenous injection of M3P (4 mg/kg) with either 100% IgG on their surface (top), 50% control IgG and 50% anti-VCAM-1 antibodies (middle) or 100% anti-VCAM-1 antibodies. (B) Corresponding quantification (n=4 per group).*p<0.05.

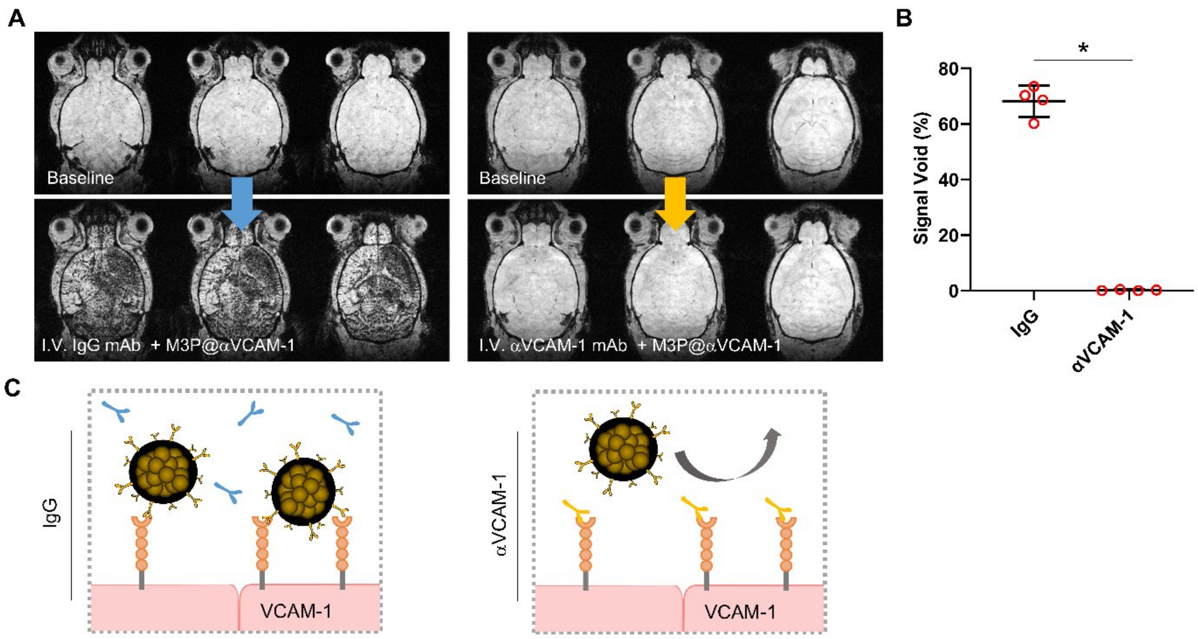

Figure S30. Saturation of M3P@αVCAM-1 binding sites with free anti-VCAM-1 monoclonal antibodies prevent M3P@αVCAM-1 binding in the inflamed brain. (A) Representative T2*-weighted images of the brain after intrastriatal injection of LPS (1.0 µg) both before and after intravenous injection of M3P@αVCAM-1 (4 mg/kg) in mice that received an intravenous injection of 100 µg of either control immunoglobulin (left) or anti-VCAM-1 monoclonal antibodies (right) 15 minutes before imaging. (B) Corresponding quantification of M3P@αVCAM-1 induced signal voids (n=4 per group). (C) Schematic representation of the findings.*$p<0.05$.

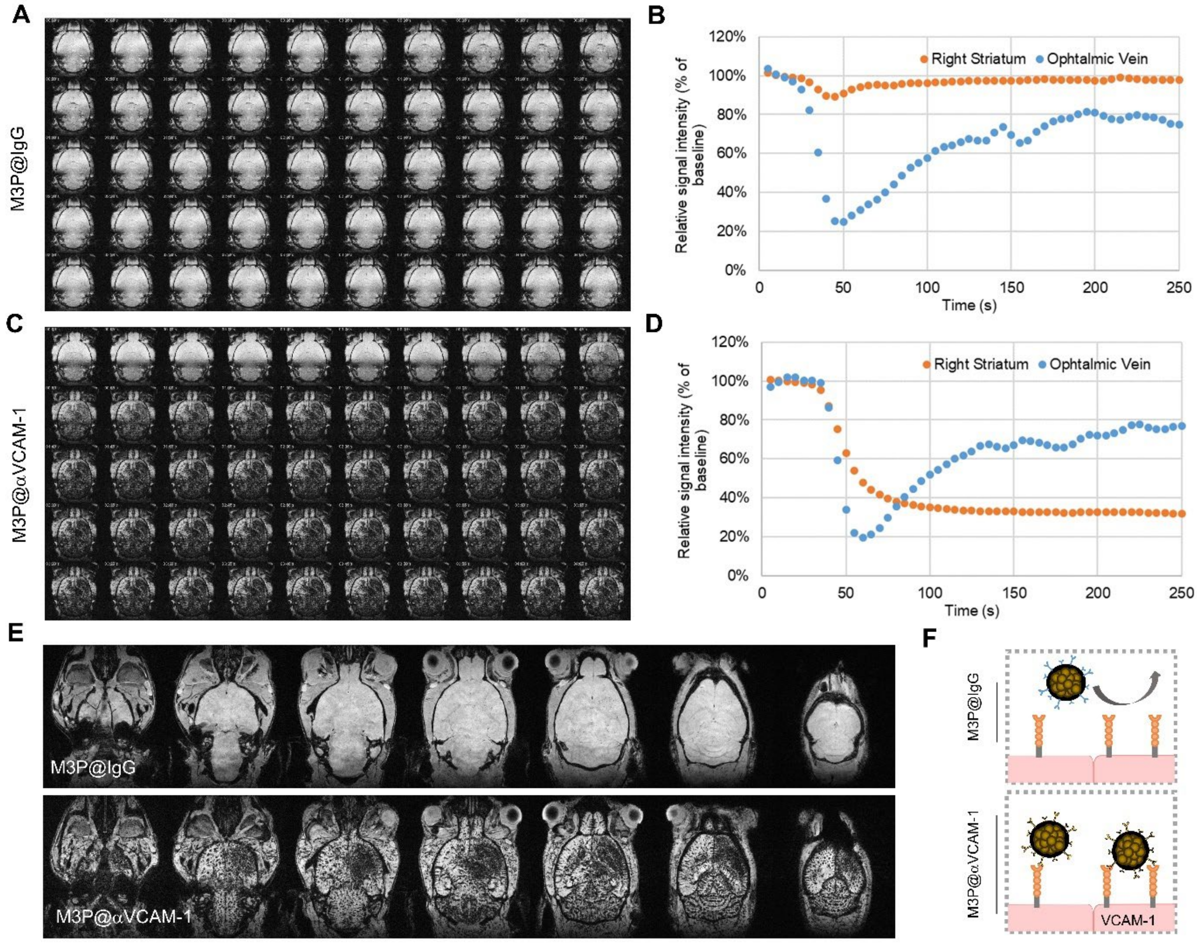

Figure S31. Analysis of the binding kinetic of M3P@IgG and M3P@αVCAM-1 in the LPS model of neuroinflammation. (A) Representative longitudinal T2*-weighted images before and after intravenous injection of M3P@IgG 24 hours after intrastriatal injection of LPS (1.0 μg). Dynamic MRI consisted of 1 image every 4 seconds. (B) Longitudinal evolution of the MRI signal in the right striatum (orange) and in the ophthalmic vein (blue). (C) Representative longitudinal T2*-weighted images before and after intravenous injection of M3P@αVCAM-1 24 hours after intrastriatal injection of LPS (1.0 μg). (D) Longitudinal evolution of the MRI signal in the right striatum (orange) and in the ophthalmic vein (blue). (E) High resolution T2*-weighted images at the end of the dynamic MRI acquisition. (F) Schematic representation of the findings. Images are representative of n=3 mice.

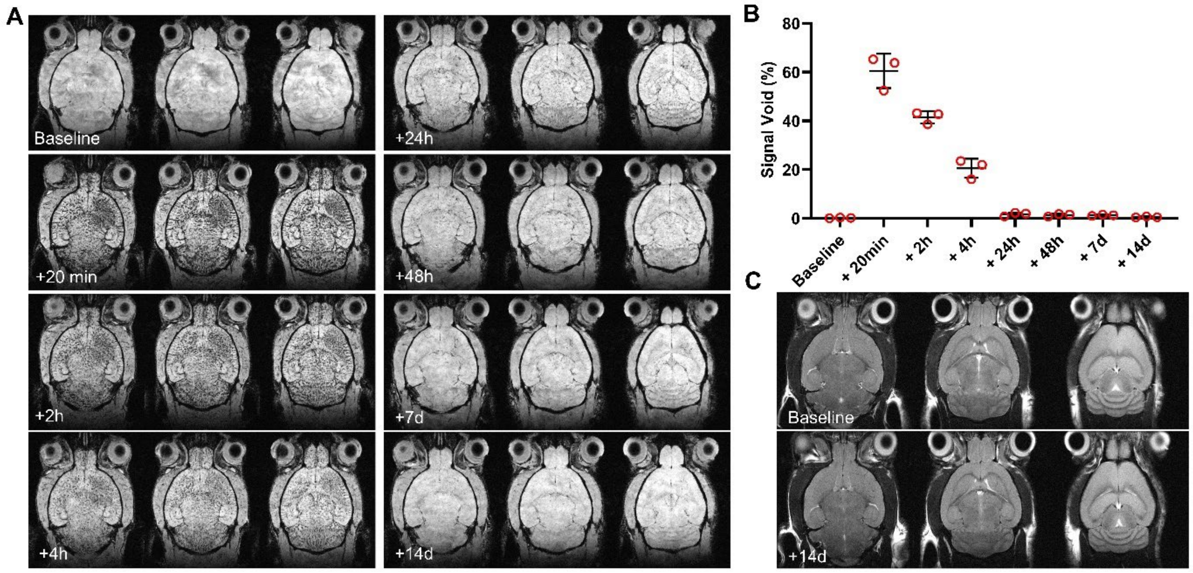

Figure S32. Longitudinal follow-up of M3P@αVCAM-1 induced signal voids reveals progressive unbinding of the particles. (A) Representative T2*-weighted images of the brain after intrastriatal injection of LPS (1.0 µg) both before and at different time points after a single intravenous injection of M3P@αVCAM-1 (4 mg/kg). (B) Corresponding quantification (n=3 mice at each time points). (C) T2-weighted images before and 14 days after a single intravenous injection of M3P@αVCAM-1 (4 mg/kg) reveal no signal abnormality in the brain.

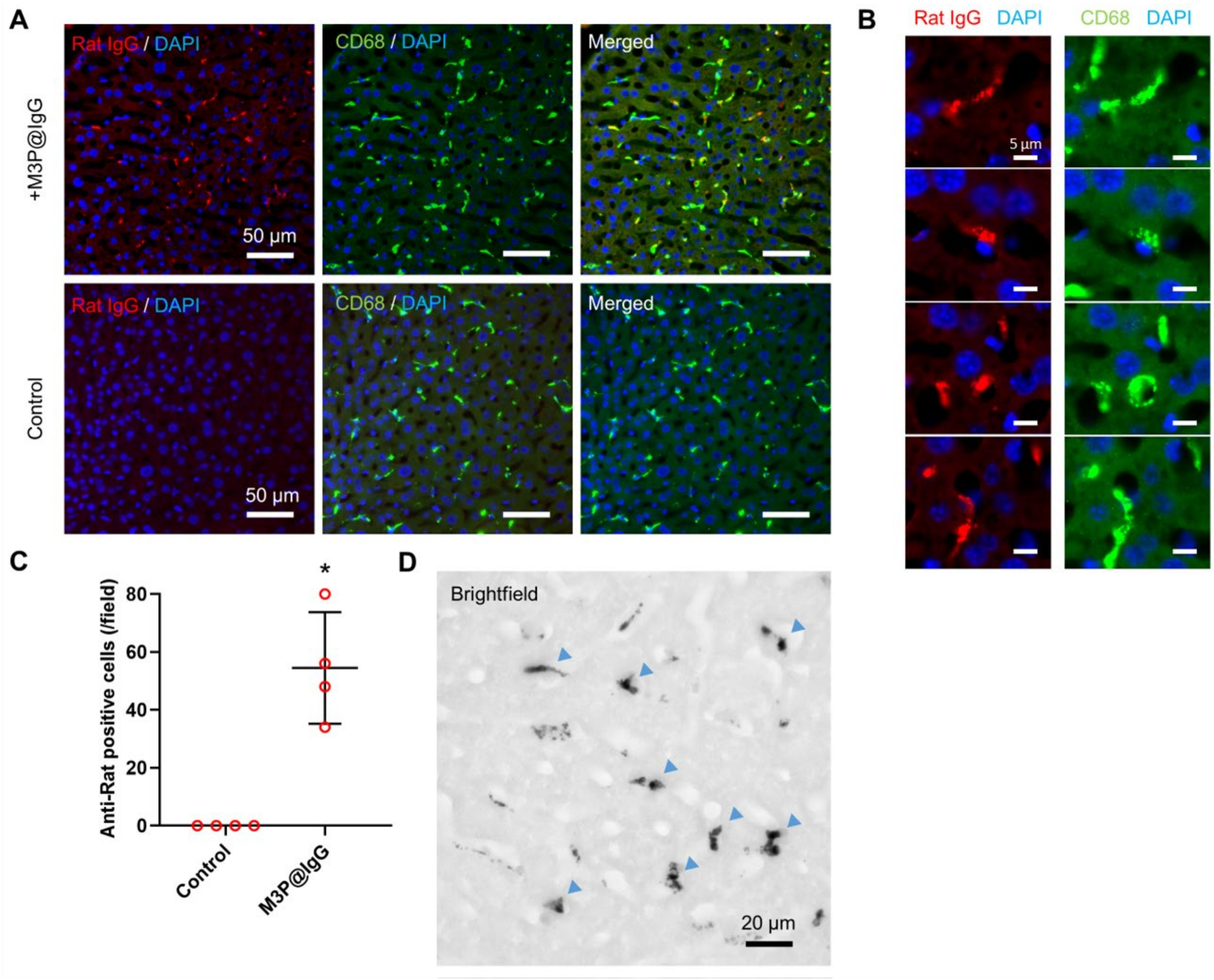

Figure S33. Unbound M3P@IgG accumulate in the macrophages of the liver. (A) Immunohistological images of the liver of mice 60 minutes after intravenous injection of M3P@IgG (4 mg/kg) or control mice. M3P@IgG are revealed by fluorescent secondary anti-rat antibodies. (B) High resolution images from A, where we can see colocalization of M3P@IgG and CD68 positive cells (macrophages) in the liver. (C) Corresponding quantification (n=4 per group). (D) Alternatively, the cells containing M3P@IgG can be seen using brightfield microscopy on which they appear black or dark brown due to the presence of PDA and magnetite nanocrystals. $*p<0.05$.

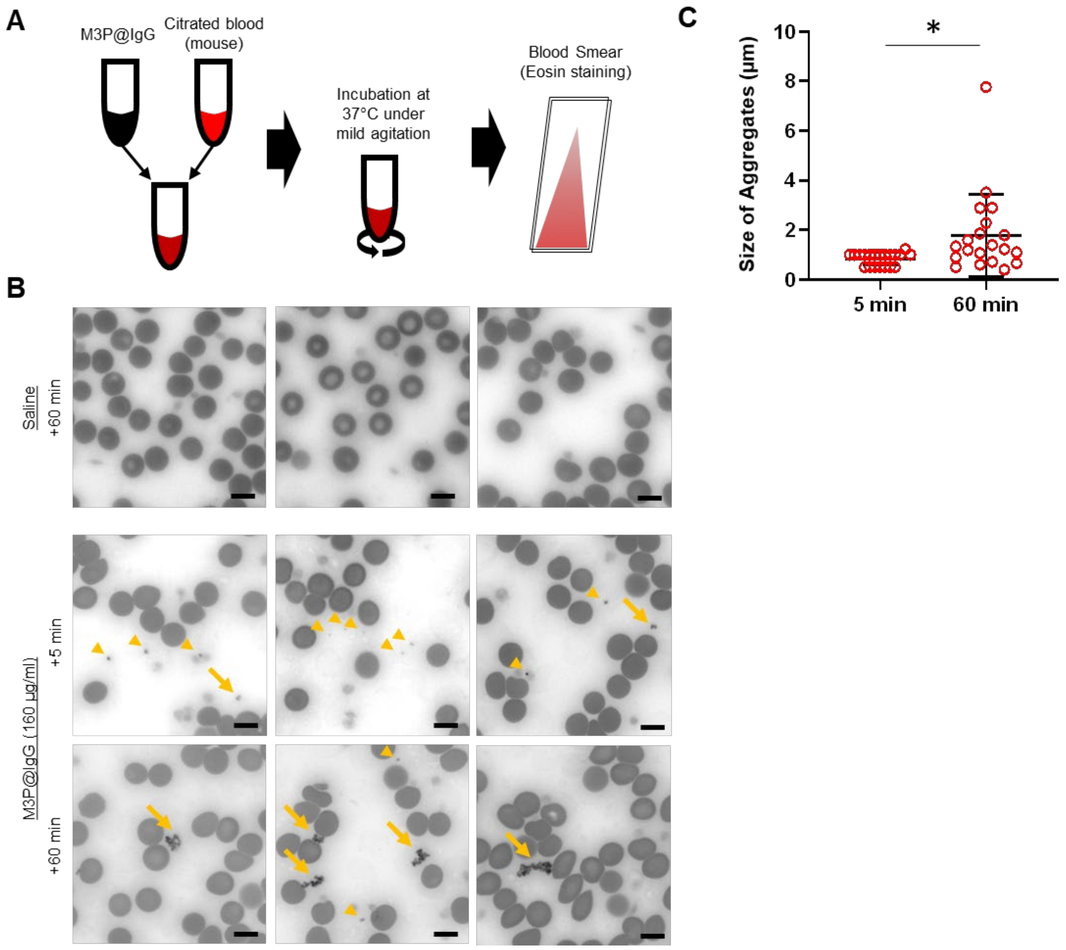

Figure S34. Stability of large 700 nm M3P@IgG in whole blood. (A) Schematic representation of the experiments. (B) Representative images from three different fields of view of blood smears in the absence of particles (top row), after 5 minutes of incubation (middle row) and after 60 minutes of incubation (bottom row) with M3P@IgG (160 µg/ml) at 37°C under mild agitation. At 5 minutes, mostly single particles were observed (arrowheads) whereas at 60 minutes, mostly aggregates were observed (arrows). Scale bar is 5 µm. (C) Corresponding quantification of the apparent size of particles at 5 minutes and 60 minutes (n=20 per group from N=3 experiments). *p<0.05.

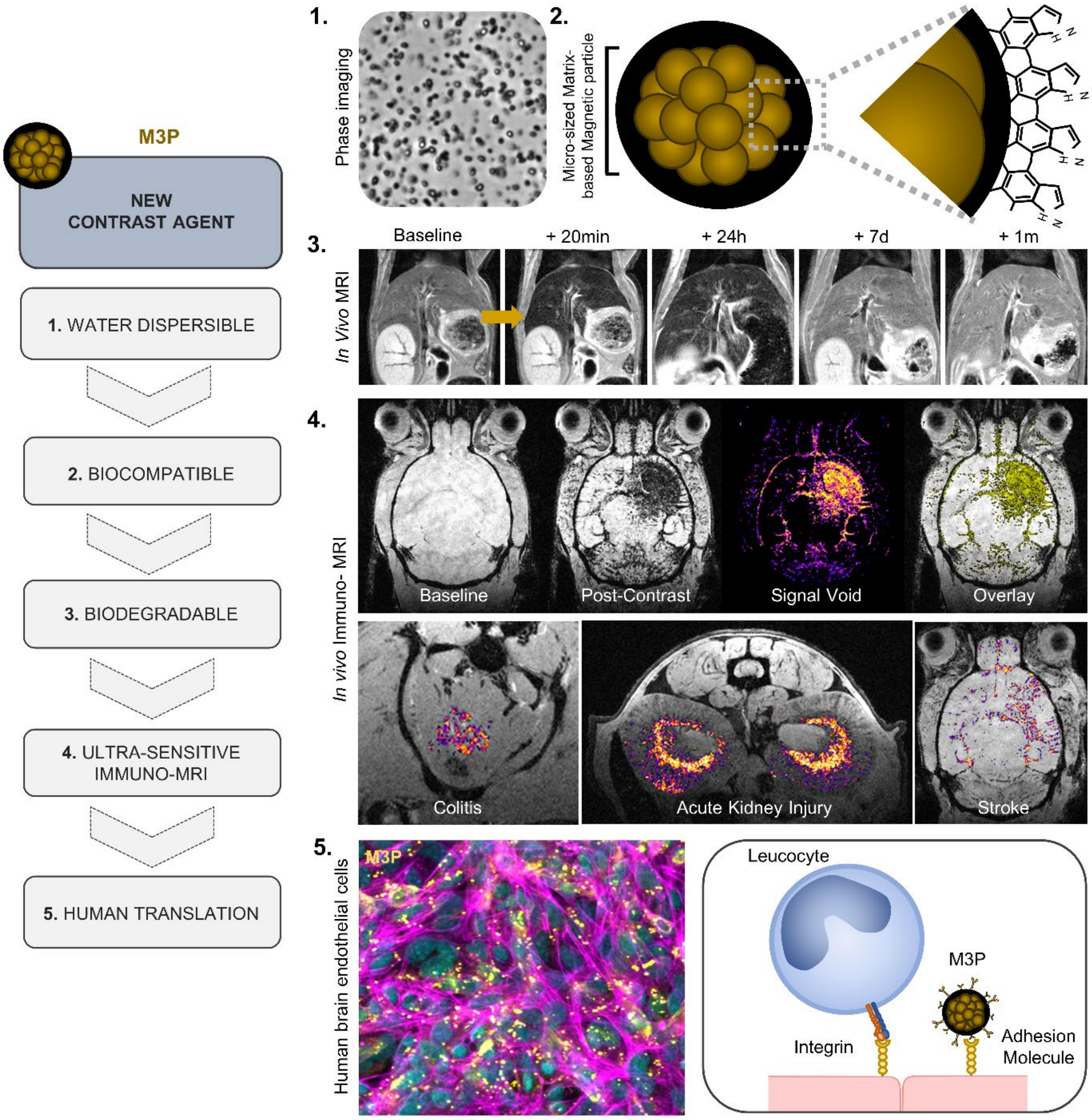

Figure S35. Graphical summary of the main results.

MOVIES LEGENDS

Movie S1. Video of water suspensions of small, medium and large M3P at 400x.

Movie S2. Video of a water suspension of large M3P at x100, x200 and x400.

Movie S3. Representative whole brain T2*-weighted images in the LPS model of neuroinflammation after intravenous injection of increasing doses of M3P@αVCAM-1.

Movie S4. Representative whole brain T2*-weighted images in the LPS model of neuroinflammation before and after intravenous injection of M3P@αVCAM-1 (4 mg/kg). 3D map of VCAM-1 expression in the brain was constructed by subtracting post-contrast images from pre-contrast images after registration.

Movie S5. Longitudinal T2*-weighted images during intravenous injection of M3P@IgG or M3P@αVCAM-1 (4 mg/kg) in the LPS model of neuroinflammation. Temporal resolution was 1 image every 4 seconds.
